# Efficacy of Digital Health Interventions for Insomnia: Systematic Review and Meta-Analysis of Randomized Controlled Trials

**DOI:** 10.2196/90905

**Published:** 2026-11-13

**Authors:** Shan Gao, Baiting Zhang, Xiaobo Li

**Affiliations:** 1 China Medical University, Shen Yang, Liao Ning, China; 2 The First Hospital of China Medical University, Nanjing North Street No. 155, Shen Yang, Liao Ning, China, 86 13998215279

**Keywords:** digital health, mobile health, insomnia, meta-analysis, systematic review

## Abstract

**Background:**

Insomnia is common, and treatments are limited by tolerability and access. Digital health interventions (DHIs) are increasingly used for sleep management, but evidence comparing multiple outcomes is scarce.

**Objective:**

This study aimed to systematically evaluate the effects of DHIs on 8 sleep outcomes, including insomnia severity, sleep efficiency, total sleep time, and sleep quality.

**Methods:**

We systematically searched PubMed, Embase, Cochrane Library, Web of Science, Scopus, CINAHL, CNKI, CBM, VIP, Wanfang, ClinicalTrials.gov, and the American Academy of Sleep Medicine’s website from inception to July 2026. Two reviewers (SG and BZ) independently screened studies, extracted data, and assessed risk of bias using the Cochrane Risk of Bias 2.0 tool. We included randomized controlled trials or controlled clinical trials comparing DHIs with any control in insomnia, without language restriction, and excluded experimental sleep deprivation, conference abstracts, protocols, and nonrandomized or noncontrolled designs. Pooled standardized mean differences (SMDs) with 95% CIs used restricted maximum likelihood with Hartung-Knapp-Sidik-Jonkman correction; 95% prediction intervals (PIs) used the Nagashima method. We also performed subgroup analyses by intervention category, comparator type, diagnostic criteria, and country income, plus sensitivity analyses, Egger tests, and GRADE (Grading of Recommendations Assessment, Development and Evaluation) assessment.

**Results:**

We included 44 randomized controlled trials (n=10,521) published between 2004 and 2026 across 14 countries. DHIs significantly improved insomnia severity (SMD −0.91, 95% CI −1.12 to −0.70), sleep efficiency (SMD 0.59, 95% CI 0.45-0.72), total sleep time (SMD 0.20, 95% CI 0.11-0.29), sleep quality (SMD −0.61, 95% CI −0.78 to −0.45), and secondary outcomes (SMDs −0.37 to −0.64). However, 95% PIs crossed the null for insomnia severity (−2.04 to 0.22), total sleep time (−0.05 to 0.46), and all secondary outcomes. Subgroup analyses showed larger insomnia severity effects in high- versus upper-middle-income countries, modality differences for sleep efficiency, and comparator-type differences for total sleep time (all *P*<.01). Egger tests suggested small-study effects; sensitivity analyses did not change direction or significance. GRADE certainty ranged from very low to moderate.

**Conclusions:**

Although DHIs showed statistically significant average effects on multiple sleep outcomes, wide PIs crossing or approaching the null, effects approaching or below Minimum Clinically Important Difference thresholds, and very low-to-moderate GRADE certainty indicate that the evidence remains exploratory. Subgroup analyses revealed effect modifications by country income, intervention modality, and comparator type, underscoring contextual factors. Unlike previous reviews focusing on single DHI formats or average statistical effects, this review provides stratified comparisons and integrates PIs and Minimum Clinically Important Difference conversion to distinguish statistical significance from clinical relevance. This paper provides an evidence synthesis framework showing that DHI benefits are context-dependent and insufficient for a standard clinical role. In practice, DHIs cannot be recommended as a standard substitute for face-to-face cognitive behavioral therapy for insomnia, although they may serve as a low-intensity option in stepped care. Large-scale, head-to-head implementation trials in diverse populations with extended follow-up are needed to define their optimal role.

## Introduction

Insomnia disorder is characterized by difficulty initiating or maintaining sleep, early-morning awakening, and associated daytime functional impairment [1]. Global epidemiological surveys indicate that approximately 10% to 15% of adults meet diagnostic criteria for insomnia disorder, while an additional 30% experience subclinical insomnia symptoms [2,3]. Chronic sleep disturbance not only contributes to daytime fatigue, cognitive decline, and emotional dysregulation but is also closely linked to the development and progression of hypertension, type 2 diabetes, depression, and cognitive decline [4-6], placing a sustained and substantial burden on individual health and health care systems. Therefore, identifying efficient and accessible insomnia interventions has become a high priority for public health and clinical practice.

Current treatment strategies for insomnia include both pharmacological and nonpharmacological approaches. Benzodiazepines and newer nonbenzodiazepine hypnotics provide rapid symptom relief; however, long-term use is associated with tolerance, dependence, and residual daytime effects [7]. Consequently, clinical guidelines consistently recommend cognitive behavioral therapy for insomnia (CBT-I) as the first-line nonpharmacological treatment for insomnia [8,9], as it addresses maladaptive sleep-related cognitions and behaviors to produce durable therapeutic gains [10]. Nevertheless, the clinical dissemination of CBT-I faces substantial barriers, including a severe shortage of trained therapists, extended treatment duration, and the need for frequent in-person visits, which collectively prevent many patients from receiving standardized care [11]. This accessibility gap underscores the urgent need for alternative service delivery models.

Against this backdrop, digital health interventions (DHIs)—enabled by smartphones, wearable devices, and internet platforms—have rapidly emerged as a scalable, low-cost, and convenient pathway for health management [12]. The World Health Organization’s (WHO’s) Global Strategy on Digital Health 2024‐2030 explicitly calls for the effective integration of DHIs into health systems and the establishment of unified evaluation frameworks [13]. DHIs have already demonstrated significant value in self-management and health promotion for chronic conditions such as diabetes, cardiovascular disease, and depression [14,15], and their potential applicability to sleep medicine warrants close attention.

In the field of sleep medicine, DHIs offer distinct technological advantages. Smartphones and internet platforms transcend temporal and geographic constraints, enabling standardized sleep intervention content to be delivered anytime and anywhere, thereby partially alleviating the accessibility limitations of traditional care models [16]. Wearable devices enable continuous, objective sleep monitoring, providing a data foundation for personalized assessment and dynamic treatment adjustment [17]. In addition, interactive interfaces, gamification elements, and real-time feedback mechanisms may enhance patient engagement and adherence [18], offering novel tools for improving sleep health.

However, DHIs for insomnia differ substantially in their delivery platforms, levels of human support, and mechanisms of action, and these differences may translate into variable treatment effects [19]. These variations underscore the need for stratified analyses by intervention type when evaluating the overall efficacy of DHIs on insomnia outcomes. Given the absence of an internationally established classification framework that categorizes DHIs specifically along the dimension of human involvement, we developed a 3-category, researcher-derived typology, drawing on multiple authoritative sources and based on 2 core dimensions: the degree of human involvement (ie, whether and how therapist or coach support is incorporated) and technological modality (ie, the delivery platform and mode of presentation). Specifically, the WHO defines mobile health as medical practice supported by mobile devices and eHealth as the use of information and communication technologies to promote health [20], providing standardized terminology for the delivery platforms—mobile apps and web-based platforms—that underpin our “autonomous self-management” subgroup. The taxonomy of digital mental health interventions proposed by Pineda et al [21] distinguishes interventions by whether they are “consumable” (ie, requiring human resources) versus “nonconsumable” (fully automated), classifying them as “self-help with human guidance” (type 3) or “fully automated self-help” (type 4). This distinction maps directly onto our “remote interaction” and “autonomous self-management” categories. Similarly, the French National Authority for Health (Haute Autorité de Santé), in its 2021 functional classification of digital solutions, identifies “autonomy”—defined as whether a digital solution operates with or without human intervention to deliver therapeutic actions—as a core classificatory dimension [22]. In addition, the WHO’s 2023 report on Emerging Technologies and Scientific Innovation highlights the growing role of virtual reality and other emerging technologies in health apps [23], providing terminological justification for our separate “emerging technologies” subgroup. Drawing on these frameworks, we categorized the included interventions into three subgroups: (1) autonomous self-management (web-based or mobile app interventions delivering standardized, self-guided content without direct therapist involvement), (2) remote interaction (digital tools combined with periodic therapist or nurse support and feedback), and (3) emerging technologies (predominantly virtual reality or biofeedback interventions operating primarily through sensory immersion rather than cognitive restructuring).

Clinicians therefore need to know whether treatment effects differ meaningfully across these modalities. However, existing systematic reviews have focused on single DHI formats and have often failed to evaluate multiple sleep outcomes—such as sleep efficiency or wake after sleep onset—or to compare effects across modalities, limiting their clinical usefulness. For example, Linardon et al [24] examined stand-alone mobile app interventions for sleep improvement without comparing them against other DHI formats, Cerolini et al [25] focused exclusively on virtual reality and provided a quantitative synthesis of its sleep effects but did not include other modalities, and Huang et al [26] included multiple DHI types but reported only insomnia severity and sleep quality, thus providing an incomplete picture of multidimensional sleep improvement. Collectively, these gaps indicate that no systematic comparison of different DHI modalities across multiple sleep outcomes in insomnia patients has yet been performed.

Given these considerations, we conducted a systematic review and meta-analysis of randomized controlled trials (RCTs) to comprehensively evaluate the effects of different DHI modalities on multidimensional sleep outcomes in individuals with insomnia. We used subgroup analyses to explore the potential influences of intervention functional category, comparator type, insomnia diagnosis criteria, and country income level, and we calculated 95% prediction intervals (PIs) to estimate the range of effects likely to be observed in future clinical settings, thereby providing evidence to inform individualized intervention selection.

The primary objective of this study was to determine whether DHIs, compared with nondigital controls, could significantly improve insomnia severity, sleep efficiency, total sleep time, sleep quality, and other outcomes, totaling 8 sleep-related measures, and to assess whether these improvements were clinically meaningful. We hypothesized that DHIs would demonstrate significant benefits across all 8 outcomes, but that the magnitude of these effects would be modified by intervention modality (autonomous self-management, remote interaction, or emerging technologies), comparator type, and country income level, with remote interaction and high-income settings yielding larger effect sizes.

## Methods

### Design

This systematic review and meta-analysis follows the methodological guidance outlined in the Cochrane Handbook for Systematic Reviews of Interventions [27] and is reported in accordance with the PRISMA (Preferred Reporting Items for Systematic Reviews and Meta-Analyses) 2020 expanded checklist [28] and the PRISMA-S (Preferred Reporting Items for Systematic Reviews and Meta-Analyses literature search extension) guideline [29]. The completed PRISMA 2020 abstract checklists, PRISMA 2020 expanded checklists, and PRISMA-S checklist are provided in Checklists 1-3, respectively. The protocol was registered on the PROSPERO (International Prospective Register of Systematic Reviews) platform under registration number CRD420251207504, and no deviations from the protocol occurred.

### Eligibility Criteria

The eligibility criteria were defined using the PICOS framework, and studies meeting all of the following criteria were included in this review.

For the population criterion, we included patients meeting diagnostic criteria for insomnia disorder according to standardized diagnostic manuals (eg, *Diagnostic and Statistical Manual of Mental Disorders* [Fifth Edition]; *International Classification of Sleep Disorders, Third Edition*; and *International Classification of Diseases, 11th Revision*) [1,30,31] or validated questionnaire cutoffs (eg, *Insomnia Severity Index* [ISI]≥8 and Pittsburgh Sleep Quality Index [PSQI]>5), as well as individuals with subthreshold insomnia. Sleep problems had to be confirmed by at least one of the following: clinical diagnostic interview, validated sleep questionnaire, or prospective sleep diary.

For the intervention criterion, we included studies evaluating any form of DHI for insomnia, including but not limited to web-based platforms, mobile apps, text messaging programs, remote monitoring systems, virtual reality, and other technology-delivered interventions targeting sleep improvement. No restrictions were placed on intervention duration, frequency, or delivery setting.

For the comparator criterion, we classified control conditions into 2 categories: passive controls (eg, waiting list and treatment as usual) and low-intensity active controls (eg, sleep hygiene education and face-to-face counseling).

For the outcome criterion, primary outcomes were insomnia severity measured by the ISI [32], sleep efficiency and total sleep time measured by sleep diary or actigraphy, and sleep quality measured by the PSQI [33]. Secondary outcomes included wake after sleep onset and sleep onset latency measured by sleep diary or actigraphy, dysfunctional beliefs about sleep measured by the Dysfunctional Beliefs and Attitudes about Sleep Scale [34], and daytime sleepiness measured by the Epworth Sleepiness Scale [35]. All outcomes had to be assessed using validated standardized instruments. For the study design criterion, we included only RCTs or controlled clinical trials, with no language restrictions. Exclusion criteria were experimental sleep deprivation studies in healthy volunteers; conference abstracts, editorials, study protocols, or studies without extractable outcome data; and nonrandomized or noncontrolled study designs.

### Information Sources

To comprehensively identify RCTs and controlled clinical trials evaluating the efficacy of DHIs for insomnia, we developed a systematic search strategy and reported it in accordance with the PRISMA-S guidelines [29]. The electronic databases searched included PubMed, Embase, Cochrane Library, Web of Science, Scopus, CINAHL, CNKI, CBM, VIP, and Wanfang, with search dates ranging from database inception to July 2026. Supplementary sources included ClinicalTrials.gov, the website of the American Academy of Sleep Medicine, and manual screening of reference lists of included studies and relevant systematic reviews to identify additional eligible studies.

### Search Strategy

We used a combination of subject headings and free-text terms, with search terms focusing on DHIs (eg, “Digital Health,” “Telemedicine,” “Mobile Applications,” “Virtual Reality,” and “Wearable Technology”) and insomnia (eg, “Insomnia” and “Sleep Wake Disorder”). No published search filters were used, either as originally designed or in modified form. The search strategies were developed de novo and were not substantively adapted from prior reviews, nor were they subjected to formal external peer review using the Peer Review of Electronic Search Strategies guideline. Detailed search strategies for all databases are presented in Multimedia Appendix 1.

### Selection Process and Data Collection

All records were imported into EndNote 21 for screening and duplicate removal. Two reviewers (SG and BZ) independently screened titles and abstracts against the eligibility criteria to identify potentially eligible studies. When titles or abstracts provided insufficient information for a decision, the full text was retrieved for further assessment. The same 2 reviewers then independently evaluated the selected full-text reports for eligibility. Interrater agreement was assessed using Cohen κ coefficient, with overall agreement rated as excellent (κ=0.86, 95% CI 0.78‐0.95). Disagreements were resolved through discussion or by consulting a third reviewer (XL).

In addition to the systematic database search, we performed backward and forward citation searches. Backward citation searching was conducted by manually screening the reference lists of all included studies and relevant systematic reviews. Forward citation searching was performed using Web of Science and Scopus to identify papers citing the included studies. Two reviewers (SG and BZ) independently screened the titles and abstracts retrieved through citation searching, obtained full texts for potentially eligible papers, and added them to the final analysis if they met the eligibility criteria.

### Data Extraction

Two reviewers (SG and BZ) independently extracted study data, with disagreements resolved through discussion and reexamination of the original papers. Extracted data included study characteristics (first author, publication year, country, study population, sample size, and age), intervention characteristics (intervention functional category, comparator type, insomnia diagnosis, country economic classification, intervention dose, and measurement instruments), and outcome data (primary outcomes: insomnia severity, sleep efficiency, total sleep time, and sleep quality; secondary outcomes: wake after sleep onset, sleep onset latency, dysfunctional beliefs about sleep, and daytime sleepiness). We extracted baseline and immediate postintervention measurements; all meta-analyses were performed on postintervention end point values.

### Study Risk-of-Bias Assessment

Two reviewers (SG and BZ) independently assessed the methodological quality of the included studies using the Cochrane Risk of Bias 2.0 tool (RoB 2) [36]. The RoB 2 tool covers 5 domains of bias: the randomization process, deviations from intended interventions, missing outcome data, outcome measurement, and selective reporting. Each domain was rated as “low risk of bias,” “some concerns,” or “high risk of bias.” Disagreements were resolved through discussion or by consulting a third reviewer (XL). An overall risk-of-bias judgment for each study was derived based on the domain-level assessments.

### Statistical Analysis and Meta-Analysis

#### Effect Size Calculation

We evaluated the feasibility of conducting a network meta-analysis or component network meta-analysis but did not proceed with either approach. Network meta-analysis was not pursued because the heterogeneity across intervention functional categories and the poorly connected evidence network, with limited head-to-head comparisons across intervention categories and an absence of common comparators to support reliable indirect estimation, precluded the transitivity assumption required for a valid analysis. Component network meta-analysis was also not feasible, as most primary studies described interventions only at the whole-program level and did not provide sufficiently standardized component-level details to allow reliable estimation of the independent effects of individual components. Therefore, we performed standard pairwise meta-analyses with subgroup analyses based on intervention functional category, comparator type, insomnia diagnosis criteria, and country income level.

All included studies had a single intervention group and a single control group, with no duplicate counting of participants. We calculated standardized mean differences (SMDs) with 95% CIs as the pooled effect size. Because insomnia diagnosis and treatment evaluation rely primarily on patient-reported outcomes, and subjective sleep measures (sleep diary, ISI, and PSQI) are recommended by international guidelines [30] as core outcome measures in insomnia clinical trials, we used subjective data as the primary analysis. For studies that also reported objective sleep measures, objective data were analyzed as secondary or sensitivity analyses to test the robustness of the findings.

Given the substantial clinical and methodological heterogeneity across studies in terms of intervention functional category, comparator type, insomnia diagnosis criteria, and country income level, we assumed a priori that true effect sizes might differ across studies. Therefore, all primary meta-analyses used a random-effects model with inverse-variance weighting and restricted maximum likelihood estimation for between-study variance, which has shown superior statistical performance compared with the conventional DerSimonian-Laird method in simulation studies. To more precisely control the false-positive rate and improve the accuracy of pooled estimates, we further applied the Hartung-Knapp-Sidik-Jonkman (HKSJ) correction to the random-effects model, re-estimating pooled effects and 95% CIs. All outcomes were converted to SMD before pooling to eliminate the influence of different measurement instruments or units across studies. Hedges *g* was used as the effect size metric to correct for potential small-study bias.

#### Heterogeneity Assessment and Handling

We assessed statistical heterogeneity across studies using the Cochran Q test (with *P*<.10 indicating statistical significance) and quantified it using the *I*² statistic. Of note, the *I*² statistic reflects the percentage of total variation attributable to between-study differences rather than sampling error, but it does not directly represent the extent of true effect variation across populations or settings. Therefore, whenever applicable and when the number of studies was sufficient (generally ≥4 studies), we calculated 95% PIs for the primary (overall) meta-analyses to quantify the real-world implications of heterogeneity. These PIs were calculated for the overall pooled estimates to contextualize the primary findings; they were not calculated for subgroup meta-analyses, as the reduced number of studies within subgroups would yield unstable and unreliable estimates. The PIs were computed based on the between-study variance (*τ*²) estimated from the random-effects model with HKSJ correction and the standard error of the pooled effect, using the method proposed by Nagashima et al [37], with the formula:


$$SMD\pm t_{0.975,df}\times \sqrt{SE^{2}+\tau^{2}}$$

where SMD is the pooled standardized mean difference, *t*_0.975, df_ is the critical value of the *t* distribution with k-1 degrees of freedom (k=number of studies), SE is the standard error of the pooled SMD (derived from the HKSJ-corrected model), and *τ*² is the between-study variance (estimated using restricted maximum likelihood). By calculating PI, we estimated the range within which the true effect for a similar population is likely to fall, thereby providing a more comprehensive assessment of the potential effects of DHIs. Therefore, following recent methodological guidance for rigorous meta-analyses, we prioritized the interpretation of these PIs alongside traditional heterogeneity metrics [38,39].

#### Subgroup Analyses

To explore potential sources of heterogeneity, we conducted subgroup analyses for the primary sleep outcomes. Subgroup analyses based on comparator type (passive comparator vs low-intensity active control), insomnia diagnosis criteria (standardized diagnostic system vs validated questionnaire only), and country income level (high-income vs upper-middle-income countries, dynamically classified according to the World Bank classification corresponding to each study’s publication year to ensure temporal accuracy [40]) were prespecified in the study protocol. Subgroup analysis based on intervention functional category (autonomous self-management, remote interaction, and emerging technologies) was also prespecified, with interventions assigned to these 3 groups. In the current manuscript, we further elaborated the operational definitions of these categories by drawing on established taxonomies, including the WHO Classification of Digital Interventions, Services and Applications in Health [20], the taxonomy proposed by Pineda et al [21], and the functional classification of the French National Authority for Health (Haute Autorité de Santé) [22], thereby strengthening the theoretical grounding of the subgroup classification. Nevertheless, given the absence of a universally accepted classification system specifically designed for efficacy comparisons across digital intervention modalities, we positioned this subgroup analysis as exploratory and hypothesis-generating, aiming to investigate patterns of differences and generate hypotheses for future research rather than to establish the causal superiority of any single modality.

To ensure reproducibility, the same 2 reviewers independently assigned each of the 44 included studies to 1 of the 3 prespecified groups based on the operationalized criteria. Interrater agreement was almost perfect (κ=0.88, 95% CI 0.70‐1.00), and disagreements were resolved through consensus discussion.

In addition, for studies that reported both subjective and objective sleep outcomes, we performed separate comparative analyses; for those reporting only subjective outcomes, we performed separate pooling. We also tested the statistical significance of subgroup differences. To evaluate the robustness of the pooled results, we conducted sensitivity analyses by sequentially excluding high-risk studies (based on RoB 2 assessments) and by using fixed-effect models as an alternative.

#### Publication Bias and Consistency Assessment

Two reviewers (SG and BZ) independently screened records and extracted data; interrater agreement was assessed using Cohen κ statistic. For the primary sleep outcomes, we constructed funnel plots and combined them with Egger tests to assess small-study effects. Funnel plot symmetry was evaluated by visual inspection in conjunction with Egger tests (*P*<.10 suggesting asymmetry, reflecting the lower statistical power of these tests in meta-analyses with limited study numbers). When a subgroup contained fewer than 10 studies, small-study effect test results were interpreted cautiously. All tests were 2-sided, with *P*<.05 considered statistically significant. Initial meta-analyses, subgroup analyses, sensitivity analyses, HKSJ correction, PI calculation, and Egger tests were performed using R (version 4.6.1; R Foundation for Statistical Computing).

#### Certainty of Evidence Assessment

We used the GRADE (Grading of Recommendations Assessment, Development and Evaluation) system to rate the certainty of evidence for each outcome, classifying it as high (⊕⊕⊕⊕), moderate (⊕⊕⊕◯), low (⊕⊕◯◯), or very low (⊕◯◯◯), representing our confidence in the effect estimates. Under the GRADE framework, evidence was downgraded on 5 domains: risk of bias (assessed using the RoB 2 tool), inconsistency (*I*²≥50% rated as serious and *I*²≥75% rated as very serious, based on Cochrane Handbook thresholds), indirectness, imprecision (95% CI crossing the null), and publication bias (indirectly assessed through funnel plots and Egger tests for small-study effects). Each domain could be downgraded by 1 level for serious concerns or by 2 levels for very serious concerns. RCTs started at high certainty. Final quality ratings were determined after downgrading.

## Results

### Study Selection

The database search yielded 26,259 records. After removing 23,174 duplicate records, 3085 records were screened by title and abstract. We excluded 2916 irrelevant records, retrieved 169 full-text papers for assessment (of which 51 were unavailable), and evaluated 118 full texts in detail. After excluding studies with nonrelevant interventions (n=19), unrelated topics (n=37), no target outcomes (n=5), non-RCT designs (n=7), and duplicates (n=6), 44 studies were finally included in the meta-analysis. In addition, among 268 records identified through other methods (eg, backward and forward citation searches), 8 full-text papers were requested, 4 entered eligibility assessment, but all were excluded because they did not involve DHIs or insomnia, resulting in no additional inclusions from this pathway. The screening process is detailed in the PRISMA flow diagram (Figure 1).

**Figure 1.**
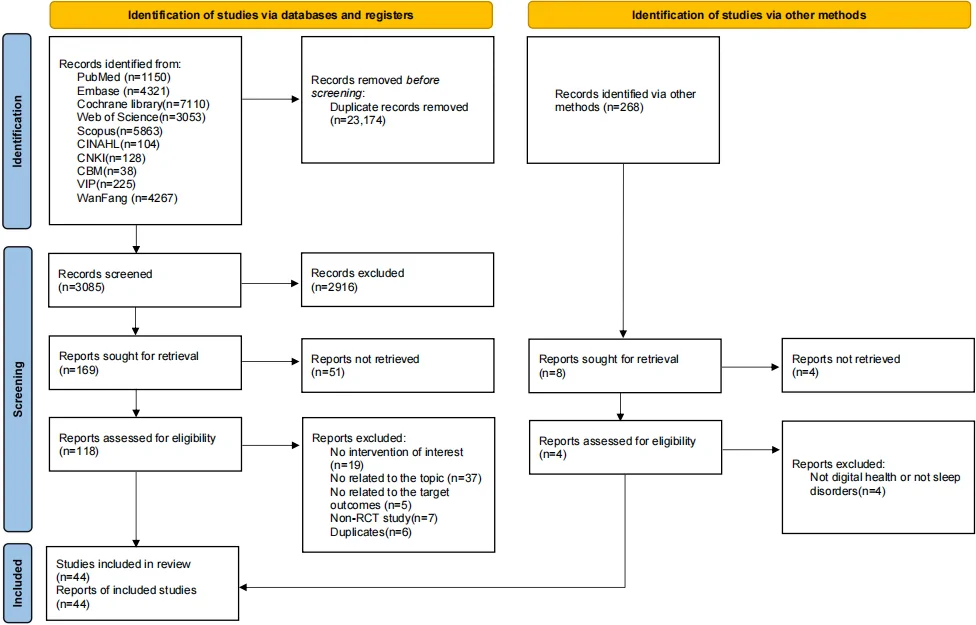
PRISMA (Preferred Reporting Items for Systematic Reviews and Meta-Analyses) flow diagram. Non-RCT: non–randomized controlled trial.

### Study Characteristics

This systematic review included 44 RCTs [10,11,16-18,41-79]. The characteristics, intervention content, and outcomes of the included studies are summarized in Table 1. Sample sizes ranged from 44 to 1721 participants, with a total of 10,521 participants across all studies. The included studies were published between 2004 and 2026 and were conducted across 14 countries. Nine studies were from China [17,18,45,47,52-54,58,59], 7 from the United States [42,51,55,65,70,73,77], 5 from the Netherlands [41,72,74,75,80], 4 from Germany [11,43,49,57], 3 from South Korea [44,46,50], 3 from Sweden [48,61,79], 3 from the United Kingdom [62,67,76], 3 from Australia [16,56,68], 2 from Japan [60,64], and 1 each from Switzerland [69], Denmark [71], Norway [63], Canada [78], and Iran [66]. All included studies were RCTs.

**Table 1.** Characteristics of included randomized controlled trials evaluating digital health interventions for sleep disorders (abridged edition; N=44). Outcome indicators: a:Insomnia Severity Index (ISI); b: sleep efficiency (SE); c: total sleep time (TST); d: Pittsburgh Sleep Quality Index (PSQI); e: sleep onset latency (SOL); f: wake after sleep onset (WASO); g: Dysfunctional Beliefs and Attitudes about Sleep (DBAS); h: Epworth Sleepiness Scale (ESS).

First author (year)	Age (years)		Country	Participants	Number of participants		Intervention functional category	Comparator type	Methodological quality	Insomnia diagnosis	Economic classification	Intervention dose	Measuring tools	Outcome indicator
	IG^a^	CG^b^			IG	CG								
Kater et al [43], 2026	46.32 (10.80)	48.62 (11.79)	Germany	Insomnia (ISI^c^ ≥8)	191	72	Autonomous self-management	Passive comparator	Some concerns	Validated questionnaire only	HIC^d^	7 sessions over 7 weeks	ISI	a
Kabbani et al [44], 2026	37.56 (11.80)	39.84 (13.15)	South Korea	Insomnia (DSM-5^e^, ISI ≥8)	27	25	Autonomous self-management	Low-intensity active control	Low risk of bias	Standardized diagnostic system	HIC	8 sessions over 6 weeks	ISI, PSQI^f^, DBAS-16^g^, ESS^h^	adgh
Wang et al [18], 2025	30.08 (4.00)	31.01 (3.69)	China	Insomnia (ISI ≥8)	80	80	Autonomous self-management	Passive comparator	Low risk of bias	Validated questionnaire only	UMIC^i^	Daily practice over 6 weeks	ISI, Sleep Diary, PSQI, ESS	abcdefh
Staiano et al [42], 2025	37.3 (11.5)	37.0 (9.6)	United States	Insomnia (ISI ≥11)	66	66	Autonomous self-management	Passive comparator	Some concerns	Validated questionnaire only	HIC	18 sessions over 6 weeks	ISI, Actigraphy, Sleep Diary, PSQI	abcdef
Chen et al [45], 2025	21.9 (2.0)	22.2 (1.9)	China	Insomnia (ICD-10^j^, ISI ≥15)	354	354	Autonomous self-management	Low-intensity active control	Low risk of bias	Standardized diagnostic system	UMIC	6 sessions over 6 weeks	ISI, PSQI, Sleep Diary, DBAS, ESS	abcefg
Park et al [46], 2025	39.8 (12.2)	37.7 (12.6)	South Korea	Insomnia (DSM-5, ISI ≥8)	33	35	Autonomous self-management	Low-intensity active control	Low risk of bias	Standardized diagnostic system	UMIC	6 sessions over 6 weeks	ISI, Sleep Diary, DBAS-16	abcefg
Chan et al [47], 2025	36.7 (14.1)	36.0 (13.8)	Hong Kong, China	Insomnia (ISI 8‐21)	179	179	Autonomous self-management	Passive comparator	Some concerns	Validated questionnaire only	HIC	7 sessions over 7 weeks	ISI, PSQI, DBAS-16	adg
Jansson-Fröjmark [48], 2025	45.9 (8.7)	48.4 (11.8)	Sweden	Insomnia (DSM-5, DSISD^k^/MINI^l^)	32	32	Remote interaction	Passive comparator	Some concerns	Standardized diagnostic system	HIC	5 sessions over 6 weeks	ISI, Sleep Diary	acef
Ell et al [49], 2024	39.4 (11.9)	40.1 (12.5)	Germany	Shift work disorder with insomnia (MINI-DIPS^m^)	23	23	Remote interaction	Passive comparator	Some concerns	Standardized diagnostic system	HIC	6 sessions over 8 weeks	ISI, Sleep Diary, Actigraphy, DBAS-16, ESS	abcefgh
Wan et al [17], 2024	54.65 (11.43)	51.95 (9.45)	China	Insomnia (ICSD-3^n^, PSQI ≥7)	32	20	Emerging technology	Passive comparator	Some concerns	Standardized diagnostic system	UMIC	Daily practice over 6 weeks	ISI, PSQI, PSM-100A^o^ (CPC^p^)	abcde
Shin et al [50], 2024	44.1 (13.1)	40.5 (12.8)	South Korea	Insomnia (ICSD-3, ISI ≥8)	49	49	Autonomous self-management	Low-intensity active control	Low risk of bias	Standardized diagnostic system	HIC	6 sessions over 6 weeks	Sleep Diary, DBAS-16, ESS	bcefgh
Starling et al [51], 2024	62.6 (9.8)	59.8 (8.7)	United States	Insomnia (ISI >7)	38	38	Emerging technology	Low-intensity active control	Low risk of bias	Validated questionnaire only	HIC	Daily practice over 6 weeks	ISI, Sleep Diary	abcef
Specht et al [11], 2025	49.90 (14.67)	49.75 (13.55)	Germany	Insomnia (ICD-11, ISI ≥10)	149	141	Autonomous self-management	Passive comparator	Low risk of bias	Standardized diagnostic system	HIC	6 sessions over 6 months	ISI	a
Shi et al [52], 2023	64.0 (6.9)	66.0 (7.4)	China	Insomnia (DSM-5)	60	60	Emerging technology	Passive comparator	Some concerns	Standardized diagnostic system	UMIC	Daily practice over 6 days	ISI, PSQI	ad
Yang et al [53], 2023	22.6 (2.7)	22.0 (1.6)	Taiwan, China	Insomnia (ISI ≥8)	31	30	Autonomous self-management	Passive comparator	Some concerns	Validated questionnaire only	HIC	8 sessions over 8 weeks	ISI, Sleep Diary, DBAS-16	ag
Zhang et al [54], 2023	49.6 (13.0)	50.6 (14.1)	China	Insomnia (ICSD-3, ISI >14)	41	41	Autonomous self-management	Low-intensity active control	Low risk of bias	Standardized diagnostic system	UMIC	Daily practice over 6 weeks	ISI, Sleep Diary	abcef
Verlinden et al [55], 2023	26.1 (6.1)	24.3 (3.5)	United States	Insomnia (ISI >14)	40	30	Autonomous self-management	Low-intensity active control	Low risk of bias	Validated questionnaire only	HIC	6 sessions over 9 weeks	ISI, PSQI	ad
Spina et al [56], 2023	47.17 (14.32)	48.77 (14.88)	Australia	Insomnia (ISI ≥10)	57	56	Remote interaction	Low-intensity active control	Some concerns	Standardized diagnostic system	HIC	1 session +2 check-in calls over 5 weeks	ISI, Sleep Diary, Fitbit, Dreem, DBAS, ESS	acefgh
Schuffelen et al [57], 2023	44.27 (14.25)	43.20 (13.57)	Germany	Insomnia (DSM-5, ISI ≥10)	118	120	Autonomous self-management	Passive comparator	Some concerns	Standardized diagnostic system	HIC	10 sessions over 8 weeks	ISI, Sleep Diary, DBAS, ESS	agh
Werner-Seidler et al [16], 2023	14.70 (1.16)	14.74 (1.27)	Australia	Insomnia (ISI ≥10)	131	133	Autonomous self-management	Low-intensity active control	Some concerns	Validated questionnaire only	HIC	6 sessions over 6 weeks	ISI, DBAS, ESS	agh
Yang et al [58], 2023	35.6 (11.2)	35.0 (10.6)	China	Acute insomnia (DSM-5)	95	97	Autonomous self-management	Passive comparator	Low risk of bias	Standardized diagnostic system	UMIC	Daily practice over 1 week	ISI, DBAS, ESS	agh
Xu et al [59], 2022	15.8 (0.2)	15.9 (0.2)	China	Insomnia (Chinese Guideline for Insomnia)	33	44	Emerging technology	Low-intensity active control	High risk of bias	Standardized diagnostic system	UMIC	8 sessions over 4 weeks	PSQI	d
Sato et al [60], 2022	49.8 (NA)	51.0 (10.4)	Japan	Insomnia (PSQI ≥6, AIS ≥6)	106	103	Autonomous self-management	Passive comparator	Some concerns	Validated questionnaire only	HIC	4 sessions over 4 weeks	PSQI, Sleep Diary	bcde
Sunnhed et al [61], 2020	51.8 (14.5)	54.2 (14.6)	Sweden	Insomnia (DSM-5, DSISD/MINI)	73	74	Remote interaction	Passive comparator	Low risk of bias	Standardized diagnostic system	HIC	10 sessions over 10 weeks	ISI, Sleep Diary	acef
Kyle et al [62], 2020	52.5 (11.2)	52.4 (11.7)	United Kingdom	Insomnia (DSM-5, SCI)	205	205	Autonomous self-management	Passive comparator	Low risk of bias	Standardized diagnostic system	HIC	6 sessions over 6 weeks	ISI, PSQI, ESS	abh
Zweerde et al [41], 2020	51.7 (15.77)	49.4 (16.01)	Netherlands	Insomnia (≥3 nights per week, ≥3 months, daytime impairment)	69	65	Remote interaction	Passive comparator	Low risk of bias	Validated questionnaire only	HIC	5 sessions over 5 weeks	ISI, Sleep Diary	abcef
Vedaa et al [63], 2020	44.2 (13.9)	44.8 (13.7)	Norway	Insomnia (ISI ≥12)	868	853	Autonomous self-management	Low-intensity active control	Some concerns	Validated questionnaire only	HIC	6 sessions over 9 weeks	ISI, Sleep Diary	abcef
Okajima et al [64], 2020	43.4 (11.9)	40.7 (10.9)	Japan	Insomnia (ISI ≥8)	70	22	Autonomous self-management	Passive comparator	Some concerns	Validated questionnaire only	HIC	Daily practice over 2 weeks	ISI	a
Felder et al [65], 2020	33.90 (3.38)	33.20 (4.00)	United States	Insomnia (DSM-5, SCI)	105	103	Autonomous self-management	Passive comparator	Some concerns	Standardized diagnostic system	HIC	6 sessions over 6 weeks	ISI, Sleep Diary, PSQI	abcd
Rajabi Majd et al [66], 2020	36.21 (5.81)	35.29 (5.76)	Iran	Insomnia (DSM-5, ISI ≥10)	156	156	Autonomous self-management	Low-intensity active control	Some concerns	Standardized diagnostic system	UMIC	6 sessions over 6 weeks	ISI, PSQI	ad
Espie et al [67], 2019	48.4 (13.9)	47.7 (13.6)	United Kingdom	Insomnia (DSM-5, SCI ≤16)	853	858	Autonomous self-management	Low-intensity active control	Some concerns	Standardized diagnostic system	HIC	6 sessions over 12 weeks	ESS	h
Murawski et al [68], 2019	41.1 (9.8)	41.9 (10.1)	Australia	Insomnia (PSQI >5)	80	80	Autonomous self-management	Passive comparator	High risk of bias	Validated questionnaire only	HIC	Daily practice over 12 weeks	ISI, PSQI, ESS	adh
Krieger et al [69], 2019	42.2 (12.4)	45.2 (12.4)	Switzerland	Insomnia (ICSD-3, ISI ≥8)	42	21	Remote interaction	Passive comparator	Some concerns	Standardized diagnostic system	HIC	8 sessions over 8 weeks	ISI, PSQI, DBAS-16	adg
Cheng et al [70], 2019	44.5 (15.8)	45.7 (15.1)	United States	Insomnia (DSM-5, ISI >14)	358	300	Autonomous self-management	Low-intensity active control	Some concerns	Standardized diagnostic system	HIC	6 sessions over 12 weeks	ISI	a
Zachariae et al [71], 2018	53.2 (8.8)	52.9 (8.9)	Denmark	Insomnia (PSQI >5)	133	122	Autonomous self-management	Passive comparator	Some concerns	Validated questionnaire only	HIC	6 sessions over 9 weeks	ISI, Sleep Diary, PSQI	abcdef
Horsch et al [72], 2017	39 (13.0)	41 (13.9)	Netherlands	Insomnia (DSM-5, ISI ≥10)	74	77	Autonomous self-management	Passive comparator	Some concerns	Standardized diagnostic system	HIC	Daily practice over 7 weeks	Sleep Diary, ISI, PSQI, DBAS-16	abcdefg
Taylor et al [73], 2017	34.5 (8.3)	32.8 (8.1)	United States	Insomnia (DSM-5, ISI >14)	34	33	Autonomous self-management	Passive comparator	Some concerns	Standardized diagnostic system	HIC	6 sessions over 6 weeks	ISI, Sleep Diary, DBAS, ESS	abcefgh
de Bruin et al [74], 2015	15.3 (1.4)	15.9 (1.6)	Netherlands	Insomnia (DSM-4, sleep diary)	39	39	Remote interaction	Passive comparator	Some concerns	Standardized diagnostic system	HIC	6 sessions over 6 weeks	Sleep Diary, Actigraphy	bcef
Lancee et al [75], 2015	47.5 (14.4)	50.0 (13.7)	Netherlands	Insomnia (DSM-5, ISI ≥10)	36	27	Remote interaction	Passive comparator	Some concerns	Standardized diagnostic system	HIC	6 sessions over 8 weeks	Sleep Diary, ISI, DBAS-16	abcefg
van Straten et a [80], 2014	48.7 (13.8)	50.1 (11.9)	Netherlands	Insomnia (DSM-5, ISI ≥10)	59	59	Remote interaction	Passive comparator	Some concerns	Standardized diagnostic system	HIC	6 sessions over 6 weeks	Sleep Diary, PSQI	bcde
Espie et al [76], 2012	50.7 (13.8)	49.1 (13.7)	United Kingdom	Insomnia (DSM-5, SCI ≤16)	55	54	Autonomous self-management	Passive comparator	Some concerns	Standardized diagnostic system	HIC	6 sessions over 6 weeks	Sleep Diary	bcef
Ritterband et al [77], 2009	44.68 (10.61)	45.05 (11.67)	United States	Insomnia (DSM-4-TR, ISI ≥8)	22	22	Autonomous self-management	Passive comparator	Some concerns	Standardized diagnostic system	HIC	6 sessions over 9 weeks	ISI, Sleep Diary	a
Vincent et al [78], 2009	47.5 (14.4)	50.0 (13.7)	Canada	Insomnia (RDC^q^: ≥6 months ≥4 nights per week, ≥30 minutes)	59	59	Autonomous self-management	Passive comparator	Some concerns	Validated questionnaire only	HIC	5 sessions over 5 weeks	ISI, Sleep Diary, DBAS	abcefg
Ström et al [79], 2004	46.2 (11.6)	43.9 (11.4)	Sweden	Insomnia (DSM-4/ASDA^r^ criteria)	42	67	Remote interaction	Passive comparator	Some concerns	Standardized diagnostic system	HIC	5 sessions over 5 weeks	Sleep Diary, DBAS	bcefg

a IG: intervention group.

b CG: control group.

c ISI: Insomnia Severity Index.

d HIC: high-income country.

e DSM-5: *Diagnostic and Statistical Manual of Mental Disorders *(Fifth Edition).

f PSQI: Pittsburgh Sleep Quality Index.

g DBAS-16: Dysfunctional Beliefs and Attitudes about Sleep Scale-16.

h ESS: Epworth Sleepiness Scale.

i UMIC: upper-middle-income country.

j ICD-10: *International Classification of Diseases, 10th Revision.*

k DSISD: Duke Structured Interview for Sleep Disorders.

l MINI: Mini International Neuropsychiatric Interview.

m MINI-DIPS: short version of the Diagnostic Interview for Mental Disorders.

n ICSD-3: *International Classification of Sleep Disorders, Third Edition*.

o PSM-100A: Portable Sleep Monitor-100A.

p CPC: cardiopulmonary coupling.

q RDC: Research Diagnostic Criteria.

r ASDA: American Sleep Disorders Association.

### Intervention Characteristics

DHIs varied considerably across studies. Of the included studies, 30 adopted autonomous self-management approaches [11,16,18,41-46,49,52-54,56,57,59,61-67,69-72,75-77], delivering standardized self-guided intervention content—including sleep hygiene education, CBT-I modules, and sleep diaries—primarily through mobile apps or web-based platforms, with users progressing at their own pace and without direct therapist involvement; 10 used remote interaction approaches [41,48,49,56,61,69,74,75,79,80], in which study teams provided periodic guidance and feedback to participants via telephone, video, or online chat; and 4 used emerging technologies [17,51,52,59], such as virtual reality, wearable devices, and cardiopulmonary coupling assessment. Intervention durations ranged from 1 to 12 weeks.

### Risk-of-Bias Assessment

#### Randomization Process

A total of 26 studies [11,16,18,20,38,38,40,41,44-46,51-53,57-62,64-68,71] were rated as low risk because they used appropriate randomization methods and implemented adequate allocation concealment. A total of 14 studies [17,37,47-50,54,56,63,69,70,73,74,76] had some concerns because they mentioned randomization but did not clearly describe allocation concealment or blinding of group assignment. Four studies [47,48,60,77] were rated as high risk because they did not report randomization methods or provided severely insufficient descriptions. No studies were rated high risk due to the randomization process itself.

#### Deviations From Intended Interventions

A total of 19 studies [11,16,37,39-43,45,48-52,59-61,65,72] were rated as low risk because participants were blinded to group assignment or both groups received active interventions, interventions were standardized, and analyses were performed using intention-to-treat (ITT). A total of 23 studies [18,20,38,44,46,47,53,55-58,62-64,66-71,73,74,76] had some concerns because participants were aware of their group assignment, which may have influenced self-reported outcomes, although interventions were standardized and ITT analyses were used. Two studies [17,59] were rated as high risk because intervention and control groups received medications with nonstandardized types or doses, or because of serious protocol deviations.

#### Missing Outcome Data

A total of 36 studies [11,16,18,20,37,39,40,42,43,45,46,48-53,55-62,64,66-74,76] were rated as low risk because they used ITT analyses with appropriate statistical methods for handling missing data, and attrition rates were within acceptable ranges. Five studies [17,43,46,49,52] had some concerns due to high attrition or between-group imbalance without adequate ITT description. Three studies [59,68,70] were rated as high risk due to high and unbalanced dropout rates, with completer-only analyses or failure to use appropriate methods for missing data.

#### Outcome Measurement

Three studies [18,44,45] were rated as low risk for outcome measurement. A total of 39 studies [11,16,20,37,38,41-51,53-74,76] had some concerns because primary outcomes were self-reported and participants were aware of their group assignment, creating potential expectancy bias. Two studies [17,57] were rated as high risk because primary outcomes were self-reported and outcome measurement had serious deficiencies (unblinded assessment and sole reliance on self-report scales).

#### Selective Reporting

A total of 29 studies [11,16,18,20,37,39,44-46,49-53,55-58,60-64,66-70,76] were rated as low risk because they were preregistered in clinical trial registries, including the Chinese Clinical Trial Registry, ClinicalTrials.gov, the Australian New Zealand Clinical Trials Registry, the University Hospital Medical Information Network, and the Open Science Framework, clearly listed all prespecified outcomes in the methods, and fully reported between-group comparisons including nonsignificant results, with no evidence of selective reporting. A total of 14 studies [17,38,40-43,47,48,54,59,65,71,73,74] had some concerns because trial registration numbers were not identified, registration information was incomplete, or multiple comparisons or outcomes were not fully prespecified in the registered protocol; although methods and results were generally complete, the lack of adequate preregistration evidence led to a conservative rating of some concerns. One study [77] was rated as high risk due to lack of preregistration and incomplete reporting. The risk-of-bias assessments are presented in a traffic-light plot (Figure 2 [11,16-18,41-80]), a summary figure (Figure S1 in Multimedia Appendix 2), and a summary of clinical trial registration numbers (Multimedia Appendix 3 [11,16-18,41-80]).

**Figure 2.**
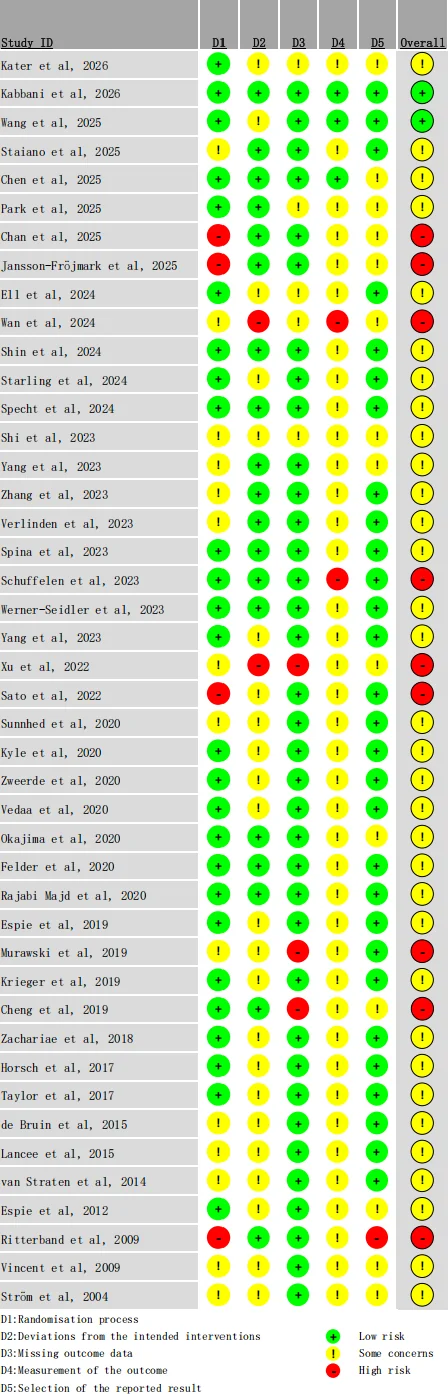
Risk-of-bias traffic-light plot for 44 randomized controlled trials using the Cochrane Risk of Bias 2.0 (RoB 2) tool, evaluating internal validity across 5 domains for digital health interventions targeting sleep disorders [11,16-18,41-80].

### Meta-Analysis Results

#### Primary Outcome Results

##### Effect of DHIs on Insomnia Severity

A total of 36 studies involving 6978 participants assessed insomnia severity using the ISI [11,16-18,20,37-44,46-53,56-61,63-68,70,72,73] (Figure 3 [11,16-18,41-49,51-58,61-66,68-73,75,77,78]). In the random-effects meta-analysis with HKSJ correction, DHIs significantly reduced insomnia severity (SMD −0.91, 95% CI −1.12 to −0.70; *I*²=86.9%, *τ²*=0.3004). However, the 95% PI calculated using the Nagashima method was wide and crossed the null (−2.04 to 0.22) [37], suggesting that a substantial proportion of individuals in future clinical practice may derive no benefit or even worsening. When converted to the ISI scale, this SMD corresponds to approximately 2.0 to 2.5 points, reaching only the lower boundary of the previously suggested minimal clinically important difference (2 to 3 points) [77-79], further underscoring the uncertainty of achieving clinically meaningful benefit at the individual level.

**Figure 3.**
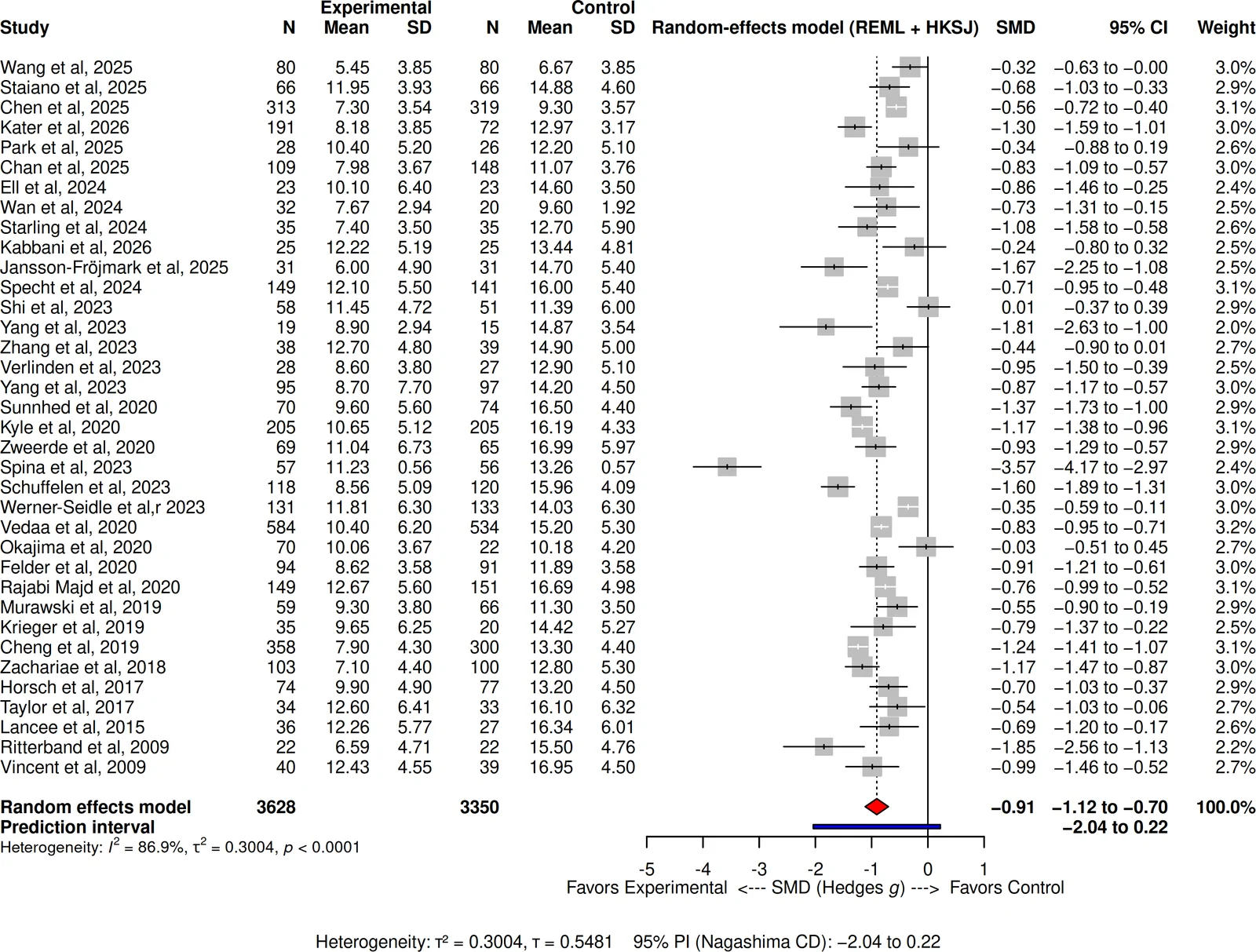
Forest plot of digital health interventions versus nondigital controls for insomnia severity [11,16-18,41-49,51-58,61-66,68-73,75,77,78]. HKSJ: Hartung-Knapp-Sidik-Jonkman random-effects model; PI: prediction interval; REML: restricted maximum likelihood; SMD: standardized mean difference.

In prespecified subgroup analyses, country income level was the only factor showing a significant interaction (*P*<.01), with high-income countries demonstrating substantially larger effects (SMD −1.03) than upper-middle-income countries (SMD −0.52). In contrast, no significant subgroup differences were observed for intervention functional category (*P*=.24), comparator type (*P*=.92), or insomnia diagnosis criteria (*P*=.39). Point estimates suggested slightly larger effects for low-intensity active controls (SMD −0.93) versus passive comparators (SMD −0.90), for remote interaction (SMD −1.40) versus autonomous self-management (SMD −0.82) and emerging technologies (SMD −0.58), and for standardized diagnostic systems (SMD −0.97) versus questionnaire-only thresholds (SMD −0.81), although none of these differences reached statistical significance. Detailed subgroup results are presented in Table 2 and Figures S2 to S5 in Multimedia Appendix 2.

**Table 2.** Subgroup analyses for insomnia severity (Insomnia Severity Index).

Subgroup variable and subgroup level	Studies, n	Sample size, n	Random-effects model SMD^a^ (95% CI)	Heterogeneity (*I*², *τ*²)	*P* value for subgroup difference
Intervention functional category					.24
Autonomous self-management	26	6130	−0.82 (−0.99 to −0.65)	*I²*=83.5%, *τ*²=0.1290	
Remote interaction	7	617	−1.40 (−2.33 to −0.47)	*I*²=91.6%, *τ*²=0.9211	
Emerging technology	3	231	−0.58 (−1.99 to 0.83)	*I*²=84.3%, *τ*²=0.2748	
Comparator type					.92
Passive comparator	25	3587	−0.90 (−1.09 to −0.71)	*I*²=80.7%, *τ*²=0.1584	
Low-intensity active control	11	3391	−0.93 (−1.54 to −0.32)	*I*²=92.9%, *τ*²=0.7379	
Insomnia diagnosis criteria					.39
Validated questionnaire only	14	2986	−0.81 (−1.05 to −0.57)	*I*²=78.4%, *τ*²=0.1150	
Standardized diagnostic system	22	3992	−0.97 (−1.29 to −0.65)	*I*²=89.6%, *τ*²=0.4399	
Country income level					<.01
High-income countries	28	5402	−1.03 (−1.28 to −0.78)	*I*²=86.7%, *τ²*=0.3267	
Upper-middle-income countries	8	1576	−0.52 (−0.76 to −0.28)	*I²*=63.9%, *τ²*=0.0533	

a SMD: standardized mean difference.

In robustness analyses, Egger test suggested small-study effects (intercept=−1.11, 95% CI −1.60 to −0.62; *P*<.01; Figure 4). Nevertheless, leave-one-out sensitivity analyses and trim-and-fill adjustments did not materially alter the direction or significance of the pooled estimate, indicating that the core findings of this study were not substantially affected by potential publication bias.

**Figure 4.**
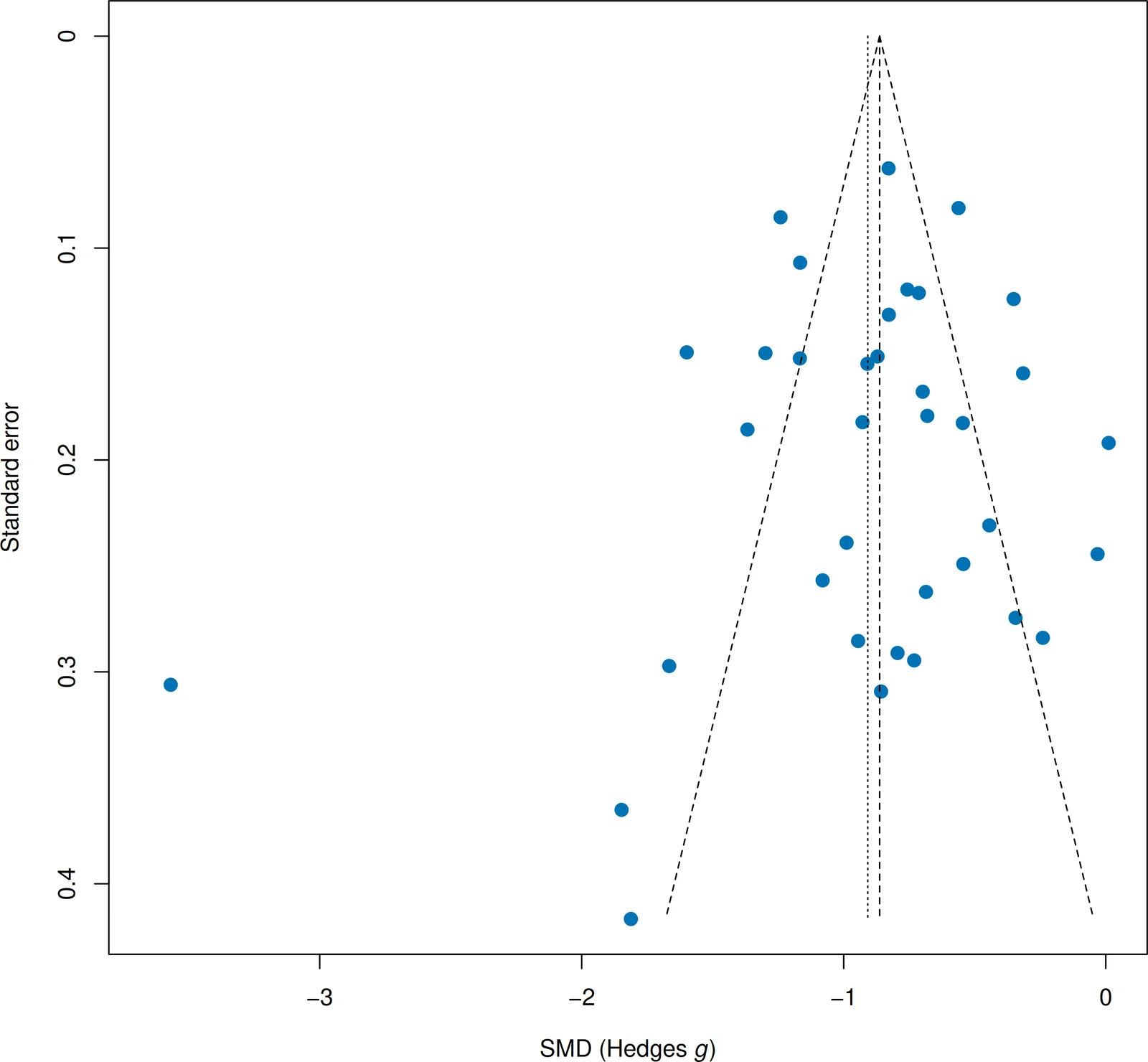
Funnel plot for assessment of small-study effects in studies reporting insomnia severity. SMD: standardized mean difference.

##### Effect of DHIs on Sleep Efficiency

A total of 23 studies involving 4107 participants assessed sleep efficiency using sleep diaries or actigraphy [17,18,20,37,40,41,44-46,49,55,57,58,60,66-71,73,74,76] (Figure 5 [17,18,41,42,45,46,49-51,54,60,62,63,65,71-76,78-80]). In the random-effects meta-analysis with HKSJ correction, DHIs were associated with a statistically significant improvement in sleep efficiency (SMD 0.59, 95% CI 0.45-0.72; *I*²=69.0%, *τ²*=0.0634). The 95% PI calculated using the Nagashima method was 0.05 to 1.13 [37], indicating that in future clinical practice, a substantial proportion of individuals may derive minimal or no meaningful improvement. When converted to absolute terms using a typical SD of 10 to 15 percentage points, this SMD corresponds to an improvement of approximately 5.8 to 8.7 percentage points; however, with the PI lower bound approaching zero and the certainty of achieving ≥85% normal sleep efficiency remaining uncertain [81], this improvement should be interpreted cautiously.

**Figure 5.**
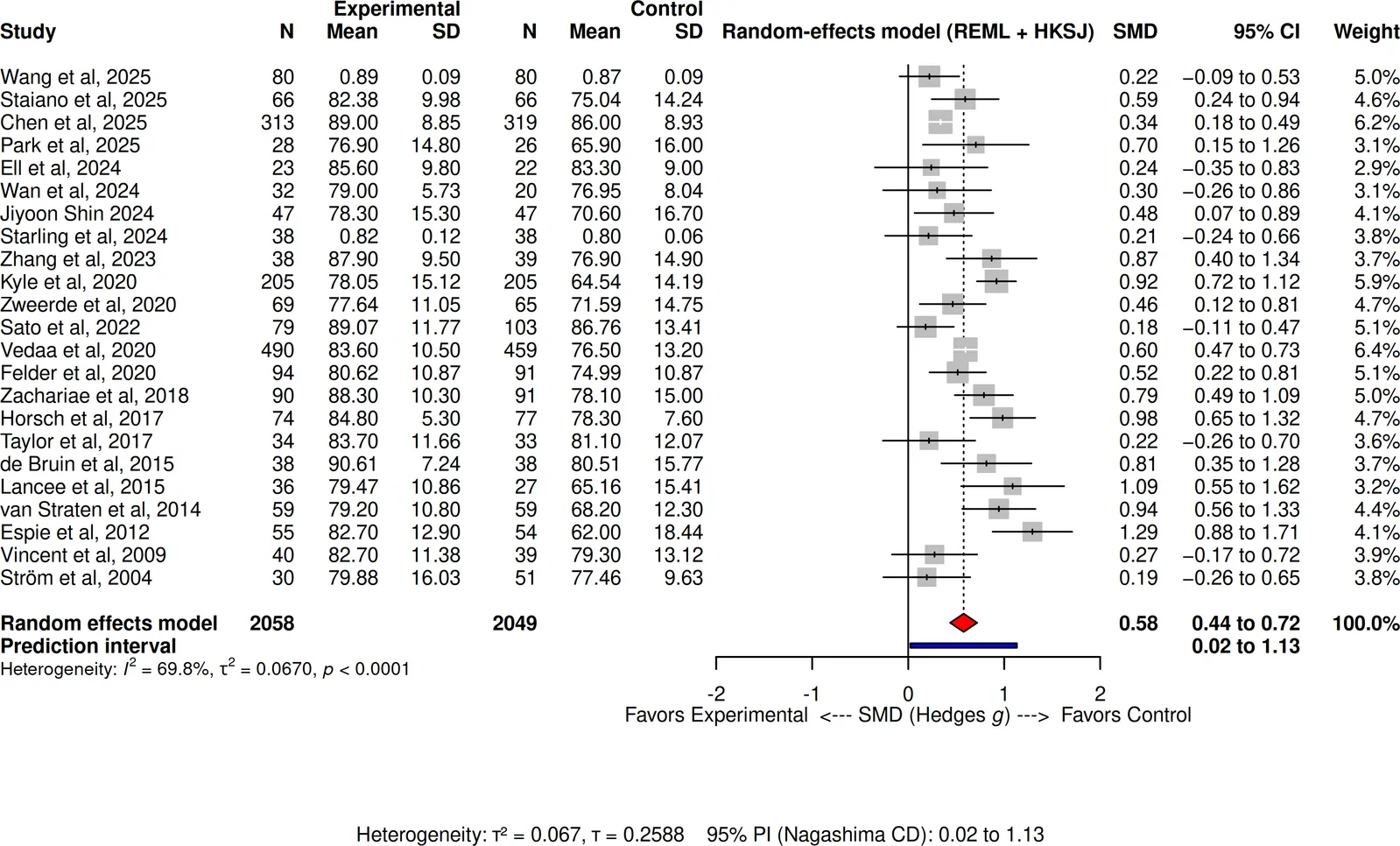
Forest plot of digital health interventions versus nondigital controls for sleep efficiency [17,18,41,42,45,46,49-51,54,60,62,63,65,71-76,78-80]. HKSJ: Hartung-Knapp-Sidik-Jonkman random-effects model; PI: prediction interval; REML: restricted maximum likelihood; SMD: standardized mean difference.

In prespecified subgroup analyses, intervention functional category was the only factor showing a significant interaction (*P*<.01), with remote interaction (SMD 0.63) and autonomous self-management (SMD 0.61) demonstrating substantially larger effects than emerging technologies (SMD 0.25). In contrast, no significant subgroup differences were observed for comparator type (*P*=.36), diagnostic criteria (*P*=.17), country income level (*P*=.16), or measurement tool (subjective vs objective; *P*=.89). Point estimates suggested slightly larger effects for passive comparators (SMD 0.61) versus low-intensity active controls (SMD 0.50), for standardized diagnostic systems (SMD 0.66) versus questionnaire-only thresholds (SMD 0.49), and for high-income (SMD 0.62) versus upper-middle-income (SMD 0.43) countries, although none of these differences reached statistical significance. A supplementary subgroup analysis of the 4 studies that used both subjective and objective measures showed comparable estimates for subjective (SMD 0.56) and objective (SMD 0.53) assessments, with no significant difference (*P*=.89). Detailed subgroup results are presented in Table 3 and Figures S6 to S10 in Multimedia Appendix 2.

**Table 3.** Subgroup analyses for sleep efficiency.

Subgroup variable and subgroup level	Studies, n	Sample size, n	Random-effects model, SMD^a^ (95% CI)	Heterogeneity (*I*², *τ*²)	*P* value for subgroup difference
Intervention functional category					<.01
Autonomous self-management	15	3462	0.61 (0.43 to 0.78)	*I*²=74.6%, *τ²*=0.0669	
Remote interaction	6	517	0.63 (0.24 to 1.01)	*I*²=59.0%, τ²=0.0757	
Emerging technology	2	128	0.25 (−0.31 to 0.80)	*I²*=0%, *τ*²=0	
Comparator type					.36
Passive comparator	17	2225	0.61 (0.43 to 0.79)	*I²*=71.2%, *τ²*=0.0826	
Low-intensity active control	6	1882	0.50 (0.29 to 0.72)	*I²*=53.6%, *τ²*=0.0183	
Insomnia diagnosis criteria					.17
Validated questionnaire only	9	2011	0.49 (0.28 to 0.69)	*I*²=62.0%, *τ*²=0.04211	
Standardized diagnostic system	14	2096	0.66 (0.47 to 0.86)	*I*²=72.8%, *τ*²=0.0736	
Country income level					.16
High-income countries	18	3132	0.62 (0.46 to 0.78)	*I*²=67.1%, *τ*²=0.0663	
Upper-middle-income countries	5	975	0.43 (0.11 to 0.75)	*I*²=41.8%, *τ²*=0.0256	
Measurement tool					.89
Subjective	4	320	0.56 (0.23 to 0.89)	*I²*=0%, *τ²*=0	
Objective	4	313	0.53 (−0.24 to 1.29)	*I²*=73.5%, *τ*²=0.1699	

a SMD: standardized mean difference.

In robustness analyses, Egger test suggested small-study effects (intercept=0.60, 95% CI 0.27-0.93; *P*<.01; Figure 6). Nevertheless, leave-one-out sensitivity analyses and trim-and-fill adjustments did not materially alter the direction or significance of the pooled estimate, supporting the robustness of our findings.

**Figure 6.**
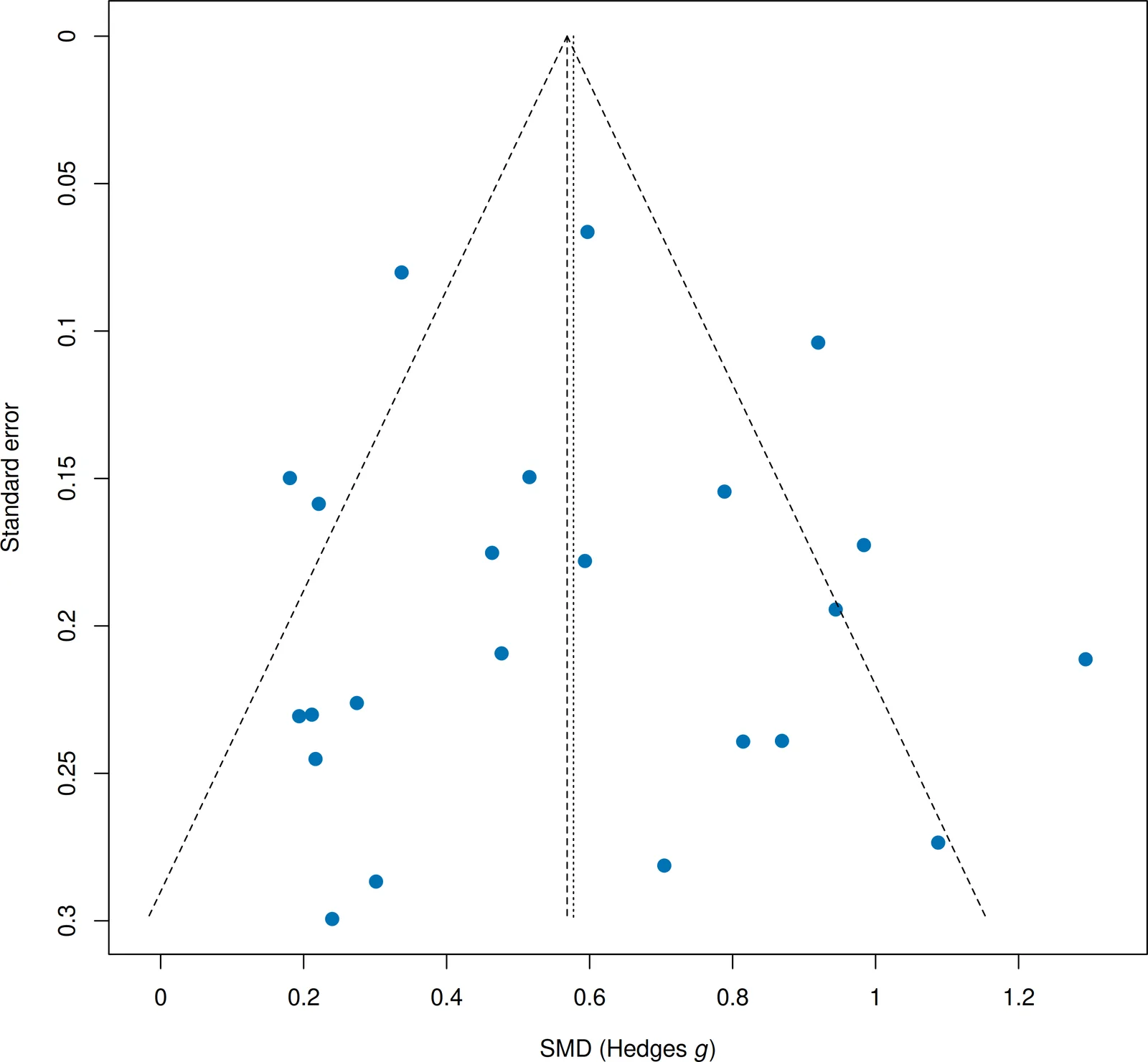
Funnel plot for assessment of small-study effects in studies reporting sleep efficiency. SMD: standardized mean difference.

##### Effect of DHIs on Total Sleep Time

A total of 25 studies involving 3998 participants assessed total sleep time using sleep diaries or actigraphy [17,18,20,37,40,41,43-46,49,51,55,56,58,60,66-71,73,74,76] (Figure 7 [17,18,41,42,45,46,48-51,54,56,60,61,63,65,71-76,78-80]). In the random-effects meta-analysis with HKSJ correction, DHIs significantly increased total sleep time (SMD 0.20, 95% CI 0.11-0.29; *I*²=31.9%, *τ²*=0.0134). However, the 95% PI calculated using the Nagashima method was −0.05 to 0.46 [37], indicating that in future clinical practice, a substantial proportion of individuals may experience no meaningful increase or even a slight decrease in total sleep time. When converted to absolute terms using a typical SD [82], this SMD corresponds to an increase of approximately 6‐12 minutes, which falls well below the widely accepted 30-minute clinical threshold for meaningful improvement [81].

**Figure 7.**
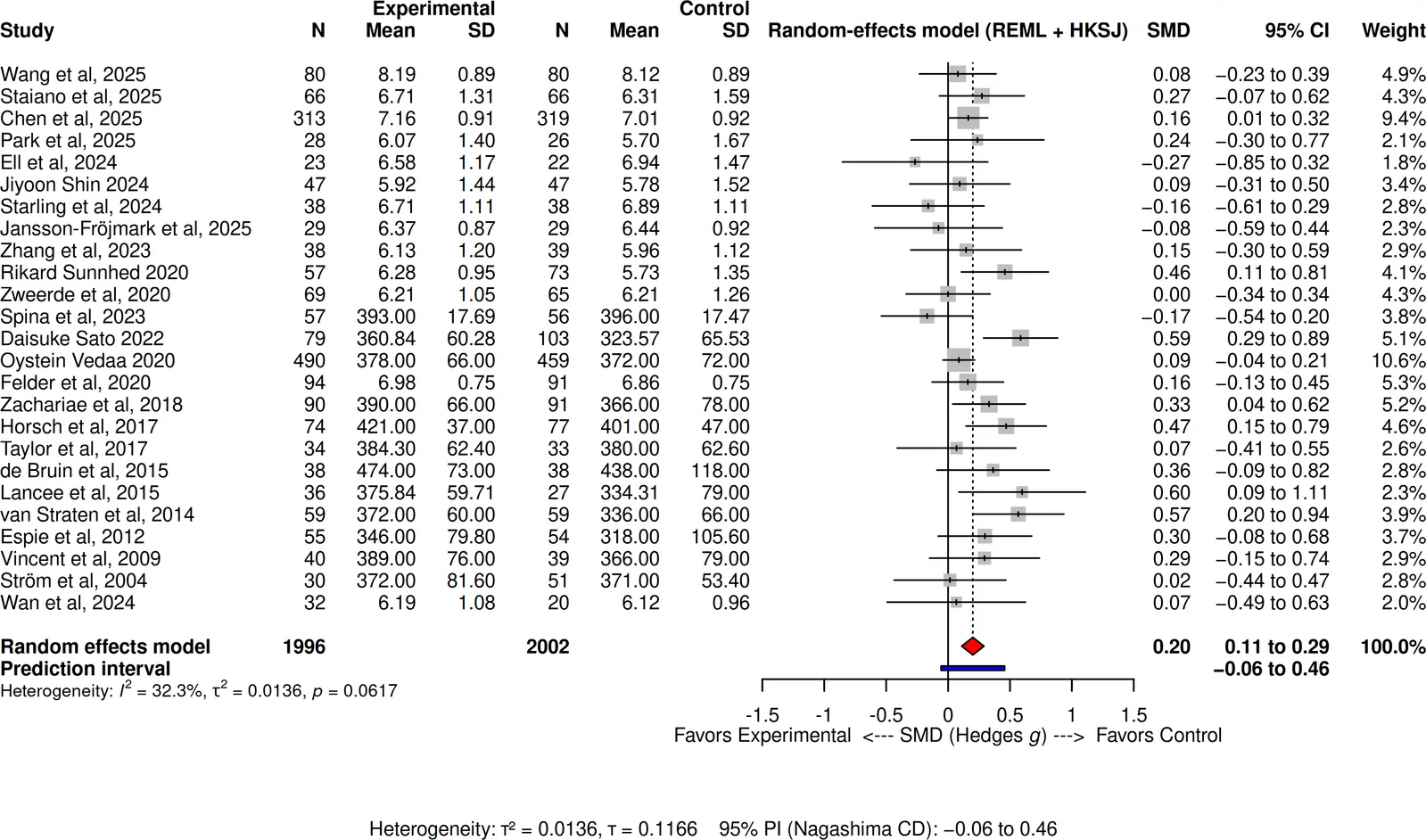
Forest plot of digital health interventions versus nondigital controls for total sleep time [17,18,41,42,45,46,48-51,54,56,60,61,63,65,71-76,78-80]. HKSJ: Hartung-Knapp-Sidik-Jonkman random-effects model; PI: prediction interval; REML: restricted maximum likelihood; SMD: standardized mean difference.

In prespecified subgroup analyses, comparator type was the only factor showing a significant interaction (*P*<.01), with passive comparators (SMD 0.27) demonstrating larger effects than low-intensity active controls (SMD 0.09). Intervention functional category showed a borderline trend (*P*=.05), with autonomous self-management (SMD 0.22) and remote interaction (SMD 0.18) demonstrating numerically larger effects than emerging technologies (SMD −0.07). In contrast, no significant subgroup differences were observed for insomnia diagnosis criteria (*P*=.66), country income level (*P*=.22), or measurement tool (*P*=.54). Point estimates suggested slightly larger effects for questionnaire-only thresholds (SMD 0.23) versus standardized diagnostic systems (SMD 0.19), and for high-income (SMD 0.22) versus upper-middle-income (SMD 0.15) countries, although none of these differences reached statistical significance. A supplementary subgroup analysis of the 5 studies that used both subjective and objective measures showed comparable estimates for subjective (SMD 0.10) and objective (SMD −0.02) assessments, with no significant difference (*P*=.54). Detailed subgroup results are presented in Table 4 and Figures S11 to S15 in Multimedia Appendix 2.

**Table 4.** Subgroup analyses for total sleep time.

Subgroup variable and subgroup level	Studies, n	Sample size, n	Random-effects model SMD^a^ (95% CI)	Heterogeneity (*I*², *τ*²)	*P* value for subgroup difference
Intervention functional category					.05
Autonomous self-management	14	3052	0.22 (0.13 to 0.31)	*I*²=11.3%, *τ*²=0.0071	
Remote interaction	9	818	0.18 (−0.07 to 0.43)	*I²*=55.7%, *τ*²=0.0571	
Emerging technology	2	128	−0.07 (−1.4 to 1.34)	*I*²=0%, *τ*²=0	
Comparator type					<.01
Passive comparator	18	2003	0.27 (0.16 to 0.38)	*I²*=24.8%, *τ*²=0.0117	
Low-intensity active control	7	1995	0.09 (0.00 to 0.19)	*I²*=0%, *τ*²=0	
Insomnia diagnosis criteria					.66
Validated questionnaire only	9	2011	0.23 (0.04 to 0.41)	*I*²=57.0%, *τ²*=0.0295	
Standardized diagnostic system	16	1987	0.19 (0.09 to 0.29)	*I²*=9.9%, *τ²*=0.0001	
Country income level					.22
High-income countries	20	3023	0.22 (0.10 to 0.34)	*I²*=44.7%, *τ*²=0.0236	
Upper-middle-income countries	5	975	0.15 (0.09 to 0.21)	*I*²=0%, *τ²*=0	
Measurement tool					.54
Subjective	5	433	0.10 (−0.24 to 0.43)	*I*²=32.2%, *τ*²=0.0229	
Objective	5	426	−0.02 (−0.42 to 0.38)	*I²*=58%, *τ*²=0.0653	

a SMD: standardized mean difference.

In robustness analyses, Egger test suggested small-study effects (intercept=0.60, 95% CI 0.27 to 0.93; *P*<.01; Figure 8). Nevertheless, leave-one-out sensitivity analyses and trim-and-fill adjustments did not materially alter the direction or significance of the pooled estimate, supporting the robustness of our findings.

**Figure 8.**
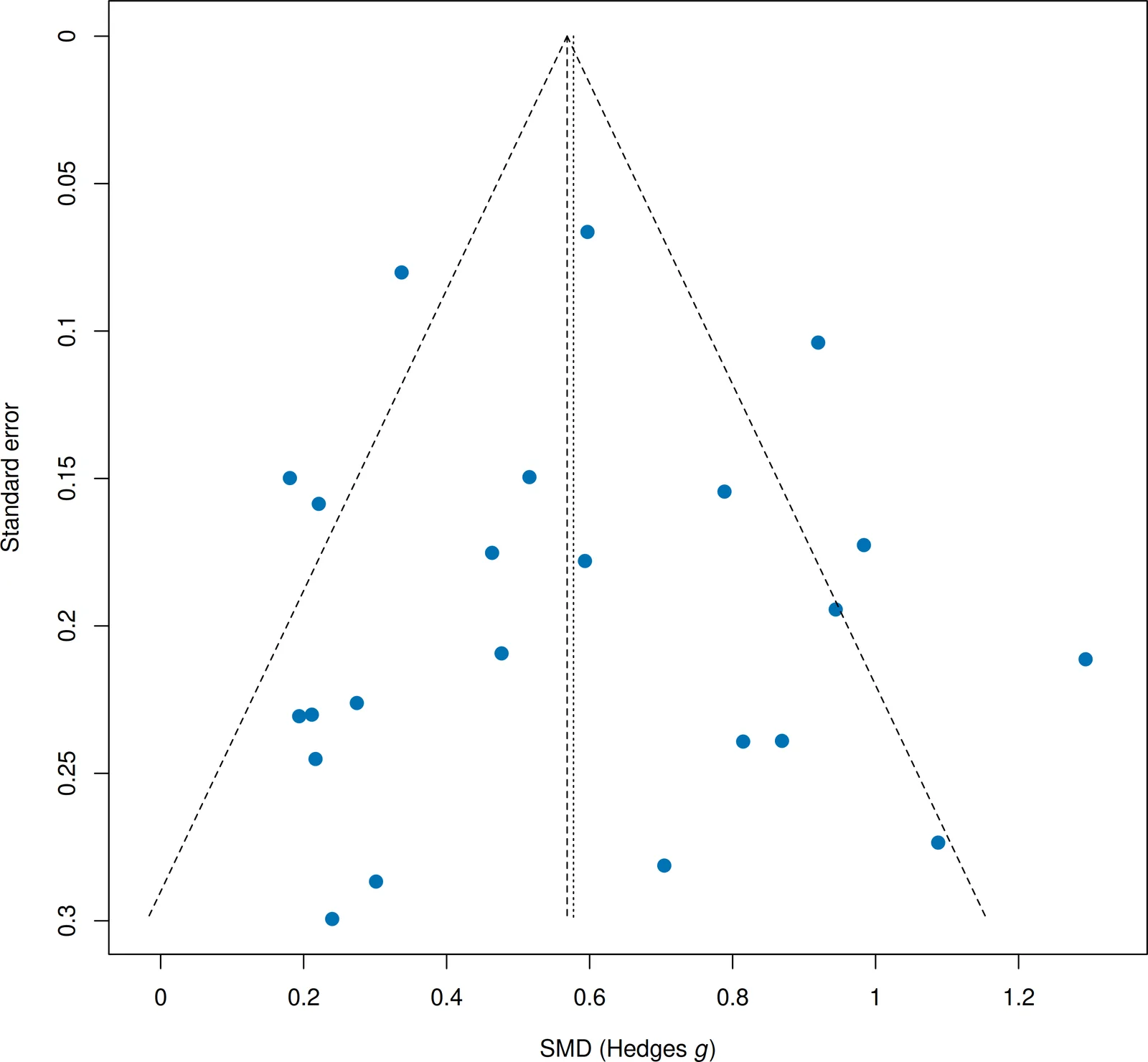
Funnel plot for assessment of small-study effects in studies reporting total sleep time. SMD: standardized mean difference.

##### Effect of DHIs on Sleep Quality

A total of 16 studies involving 2196 participants assessed sleep quality using the PSQI [17,18,42,44,47,52,55,59,60,65,66,68,69,71,72,80] (Figure 9). In the random-effects meta-analysis with HKSJ correction, DHIs were associated with a statistically significant improvement in sleep quality (SMD −0.61, 95% CI −0.78 to −0.45; *I*²=63.6%, *τ²*=0.0597). The 95% PI calculated using the Nagashima method was −1.16 to −0.07 [37], indicating that in future clinical practice, a substantial proportion of individuals may derive minimal or no meaningful improvement. The PSQI total score ranges from 0 to 21, with higher scores indicating worse sleep quality [33]. Based on a typical SD conversion, this SMD corresponds to a PSQI reduction of approximately 1.8 to 2.4 points; however, given that the PI upper bound approaches zero and the improvement falls below the previously suggested minimal clinically important difference of 2.5 to 2.7 points for the PSQI [83], this reduction should be interpreted cautiously.

**Figure 9.**
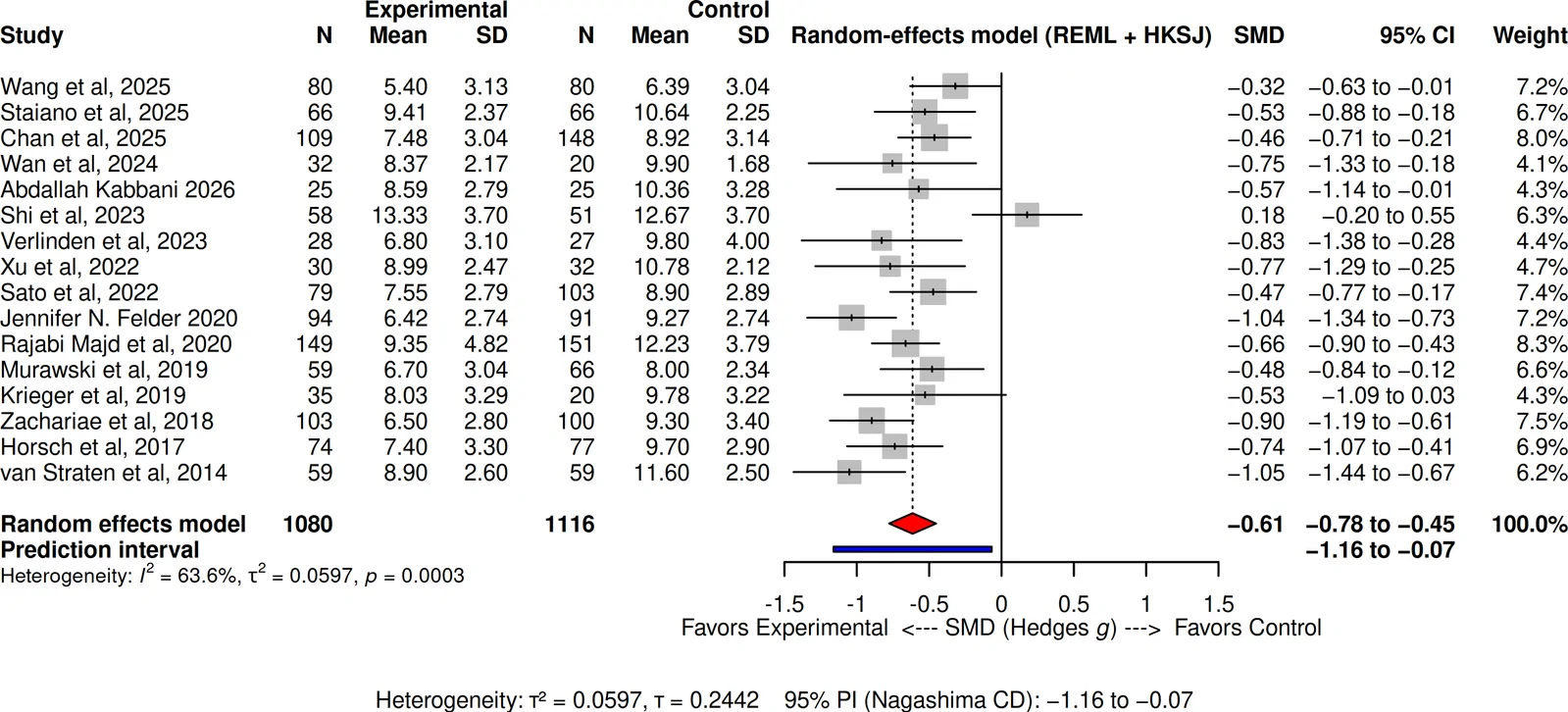
Forest plot of digital health interventions versus nondigital controls for Pittsburgh Sleep Quality Index [17,18,42,44,47,52,55,59,60,65,66,68,69,71,72,80]. HKSJ: Hartung-Knapp-Sidik-Jonkman random-effects model; PI: prediction interval; REML: restricted maximum likelihood; SMD: standardized mean difference.

In prespecified subgroup analyses, no significant subgroup differences were observed for intervention functional category (*P*=.60), comparator type (*P*=.37), insomnia diagnosis criteria (*P*=.97), or country income level (*P*=.20). Point estimates suggested slightly larger effects for remote interaction (SMD −0.83) versus autonomous self-management (SMD −0.63) and emerging technologies (SMD −0.42), for low-intensity active controls (SMD −0.69) versus passive comparators (SMD −0.59), and for high-income (SMD −0.69) versus upper-middle-income (SMD −0.45) countries. Of note, standardized diagnostic systems (SMD −0.61) and questionnaire-only thresholds (SMD −0.61) yielded identical point estimates. Detailed subgroup results are presented in Table 5 and Figures S16 to S19 in Multimedia Appendix 2.

**Table 5.** Subgroup analyses for sleep quality.

Subgroup variable and subgroup level	Studies, n	Sample size, n	Random-effects model, SMD^a^ (95% CI)	Heterogeneity (*I*², *τ*²)	*P* value for subgroup difference
Intervention functional category					.60
Autonomous self-management	11	1800	−0.63 (−0.78 to −0.48)	*I²*=46.2%, *τ²*=0.0247	
Remote interaction	2	173	−0.83 (−4.12 to 2.45)	*I²*=56.2%, *τ²*=0.0770	
Emerging technology	3	223	−0.42 (−1.80 to 0.95)	*I²*=82.9%, *τ²*=0.2563	
Comparator type					.37
Passive comparator	12	1729	−0.59 (−0.81 to −0.37)	*I²*=72.4%, *τ²*=0.0835	
Low-intensity active control	4	467	−0.69 (−0.82 to −0.56)	*I²*=0%, *τ²*=0	
Insomnia diagnosis criteria					.97
Validated questionnaire only	8	1232	−0.61 (−0.82 to −0.39)	*I²*=54.9%, *τ²*=0.0351	
Standardized diagnostic system	8	964	−0.61 (−0.92 to −0.31]	*I²*=72.4%, *τ²*=0.1014	
Country income level					.20
High-income countries	11	1513	−0.69 (−0.85 to −0.53)	*I²*=45.6%, *τ²*=0.0285	
Upper-middle-income countries	5	683	−0.45 (−0.94 to 0.05)	*I²*=76.5%, *τ²*=0.1185	

a SMD: standardized mean difference.

In robustness analyses, Egger test suggested small-study effects (intercept=−0.69, 95% CI −1.30 to −0.08; *P*=.03; Figure 10). Nevertheless, leave-one-out sensitivity analyses and trim-and-fill adjustments did not materially alter the direction or significance of the pooled estimate, supporting the robustness of our findings.

**Figure 10.**
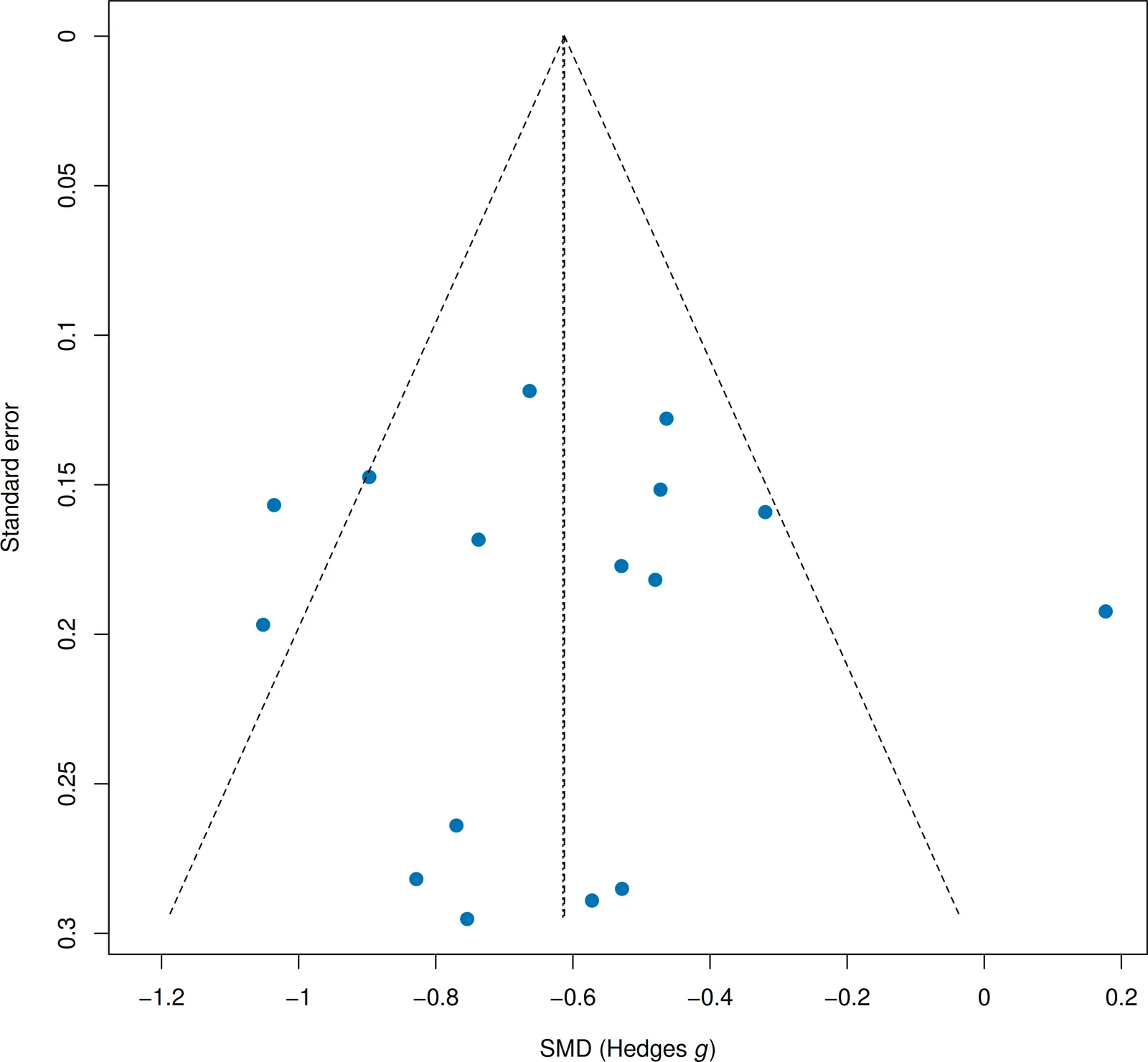
Funnel plot for assessment of small-study effects in studies reporting Pittsburgh Sleep Quality Index.

### Secondary Outcome Results

A total of 24 studies assessed sleep onset latency [17,18,20,37,40,41,43-46,49,51,55,56,58,66-71,73,74,76], 22 studies assessed wake after sleep onset [18,20,37,40,41,43-46,49,51,56,58,64,66-71,73,74], 16 studies assessed dysfunctional beliefs about sleep [16,39-42,44,45,48,51-53,67,68,70,73,74], and 12 studies assessed daytime sleepiness [16,18,39,44,45,51-53,57,62,63,68]. In random-effects meta-analyses, DHIs were associated with significant reductions in sleep onset latency (SMD −0.41, 95% CI −0.58 to −0.24; *I*²=74.3%), wake after sleep onset (SMD −0.45, 95% CI −0.66 to −0.23; *I*²=83.8%), dysfunctional beliefs about sleep (SMD −0.64, 95% CI −0.86 to −0.42; *I*²=81.2%), and daytime sleepiness (SMD −0.37, 95% CI −0.55 to −0.19; *I*²=63.6%). However, the 95% PIs for all 4 secondary outcomes crossed the null: sleep onset latency (−1.15 to 0.33), wake after sleep onset (−1.39 to 0.50), dysfunctional beliefs about sleep (−1.44 to 0.17), and daytime sleepiness (−0.87 to 0.14), indicating that future effects could range from benefit to no effect or even harm.

In supplementary subgroup analyses exploring measurement tool consistency, 5 studies that used both subjective diaries and objective actigraphy to assess sleep onset latency showed different point estimates for subjective (SMD −0.16) and objective (SMD −0.57) measures, but the subgroup difference was not statistically significant (*P*=.25). For wake after sleep onset, subjective (SMD −0.06) and objective (SMD −0.12) estimates were comparable, with no significant subgroup difference (*P*=.83).

In robustness analyses, Egger tests suggested small-study effects for all 4 secondary outcomes: sleep onset latency (intercept=−0.51, 95% CI −0.93 to −0.11; *P*=.02), wake after sleep onset (intercept=−0.63, 95% CI −1.11 to −0.15; *P*=.01), dysfunctional beliefs about sleep (intercept=−0.90, 95% CI −1.45 to −0.36; *P*=.03), and daytime sleepiness (intercept=−0.49, 95% CI −0.85 to −0.12; *P*=.01). Nevertheless, leave-one-out sensitivity analyses and trim-and-fill adjustments did not materially alter the direction or significance of any pooled estimate, supporting the robustness of our findings. Detailed subgroup results are presented in Table 6 and Figures S20 to S29 in Multimedia Appendix 2. PIs are reported for overall meta-analyses only.

**Table 6.** Summary of secondary outcomes.

Outcome	Studies, n	Sample size, n	Random-effects model, SMD^a^ (95% CI)	Heterogeneity (*I*², *τ*²)	*P* value for subgroup difference	Egger *P* value
SOL^b^						
Overall	24	3813	−0.41 (−0.58 to −0.24)	*I²*=74.3%, *τ*²=0.1206	—^c^	.02
Measurement tool					.25	
Subjective	5	433	−0.16 (−1.07 to 0.74)	*I*²=91.6%, *τ*²=0.4874	—	
Objective	5	426	−0.57 (−0.95 to −0.19)	*I²*=40.6%, *τ*²=0.0321	—	
WASO^d^						
Overall	22	3517	−0.45 (−0.66 to −0.23)	*I²*=83.8%, *τ²*=0.1965	—	.01
Measurement tool					.83	
Subjective	5	433	−0.06 (−0.81 to 0.69)	*I*²=88.4%, *τ²*=0.3266	—	
Objective	5	426	−0.12 (−0.53 to 0.28)	*I*²=55.6%, *τ*²=0.0603	—	
DBAS^e^						
Overall	16	2397	−0.64 (−0.86 to −0.42)	*I*²=81.2%, *τ*²=0.1310	—	<.01
ESS^f^						
Overall	12	3453	−0.37 (−0.55 to −0.19]	*I*²=63.6%, *τ*²=0.0466	—	.01

a SMD: standardized mean difference.

b SOL: sleep onset latency.

c Not available.

d WASO: wake after sleep onset.

e DBAS: Dysfunctional Beliefs and Attitudes about Sleep.

f ESS: Epworth Sleepiness Scale.

### Sensitivity Analysis Results

To test the robustness of our meta-analysis results, we performed leave-one-out sensitivity analyses. For all primary outcomes, exclusion of any single study did not change the direction or significance of the pooled estimates. In addition, reanalysis using a fixed-effect model yielded results consistent with the random-effects model. Taken together, these findings indicate that our meta-analysis results are generally robust to the exclusion of individual studies and the choice of modeling approach. The residual heterogeneity observed may be attributable to differences in intervention methods, measurement instruments, intervention content, patient characteristics, and technological platforms.

### Certainty of Evidence

Using the GRADE system, we rated the certainty of evidence for the prespecified outcomes. The certainty of evidence was moderate (⨁⨁⨁◯) for total sleep time, downgraded only for risk of bias (due to limitations in blinding of outcome assessment). It was low (⨁⨁◯◯) for sleep efficiency, sleep quality, sleep onset latency, and daytime sleepiness, downgraded for risk of bias and serious inconsistency (*I*²≥50%). It was very low (⨁◯◯◯) for insomnia severity, wake after sleep onset, and dysfunctional beliefs about sleep, downgraded for risk of bias and very serious inconsistency (*I*²≥75%). No outcomes were additionally downgraded for indirectness, imprecision, or publication bias (Table 7).

**Table 7.** Grading of Recommendations Assessment, Development and Evaluation certainty of evidence.

Certainty assessment							Patients, n		Effect		Certainty	Importance
Studies, n	Study design	Risk of bias	Inconsistency	Indirectness	Imprecision	Other considerations	Digital health interventions	Comparison	Relative (95% CI)	Absolute (95% CI)		
Insomnia Severity Index												
36	Randomized trials	Serious^a^	Very serious^b^	Not serious	Not serious	None	3628	3350	—^c^	SMD^d^ 0.91 lower (1.12 lower to 0.70 lower)	⨁◯◯◯ Very low^a^^,b^	Critical
Sleep efficiency												
23	Randomized trials	Serious^a^	Serious^b^	Not serious	Not serious	None	2058	2049	—	SMD 0.59 higher (0.45 higher to 0.72 higher)	⨁⨁◯◯ Low^a^^,b^	Critical
Total sleep time												
25	Randomized trials	Serious^a^	Not serious	Not serious	Not serious	None	1996	2002	—	SMD 0.20 higher (0.11 higher to 0.29 higher)	⨁⨁⨁◯ Moderate^a^	Critical
Pittsburgh Sleep Quality Index												
16	Randomized trials	Serious^a^	Serious^b^	Not serious	Not serious	None	1080	1116	—	SMD 0.61 lower (0.78 lower to 0.45 lower)	⨁⨁◯◯ Low^a^^,b^	Critical
Sleep onset latency												
24	Randomized trials	Serious^a^	Serious^b^	Not serious	Not serious	None	1902	1911	—	SMD 0.41 lower (0.58 lower to 0.24 lower)	⨁⨁◯◯ Low^a^^,b^	Critical
Wake after sleep onset												
22	Randomized trials	Serious^a^	Very serious^b^	Not serious	Not serious	None	1768	1749	—	SMD 0.45 lower (0.66 lower to 0.23 lower)	⨁◯◯◯ Very low^a^^,b^	Critical
Dysfunctional Beliefs and Attitudes about Sleep Scale												
16	Randomized trials	Serious^a^	Very serious^b^	Not serious	Not serious	None	1169	1228	—	SMD 0.64 lower (0.86 lower to 0.42 lower)	⨁◯◯◯ Very low^a^^,b^	Important
Epworth Sleepiness Scale												
12	Randomized trials	Serious^a^	Serious^b^	Not serious	Not serious	None	1717	1736	—	SMD 0.37 lower (0.55 lower to 0.19 lower)	⨁⨁◯◯ Low^a^^,b^	Important

a Some studies fail to adequately describe the implementation of randomization procedures, allocation concealment, and blinding of outcome assessors.

b High heterogeneity.

c Not available.

d SMD: standardized mean difference.

## Discussion

### Principal Findings

This systematic review and meta-analysis evaluated the efficacy of DHIs for improving sleep outcomes in individuals with insomnia. Our findings indicate that DHIs were associated with statistically significant improvements in insomnia severity, sleep efficiency, sleep quality, and several secondary outcomes, including sleep onset latency, wake after sleep onset, dysfunctional beliefs about sleep, and daytime sleepiness.

Subgroup analyses revealed several notable patterns across intervention characteristics. For sleep efficiency, remote interaction and autonomous self-management demonstrated comparable effects, which were substantially larger than those of emerging technologies. Remote interaction may address the adherence limitations of pure self-guided models through regular therapist supervision and personalized feedback [84], autonomous self-management enables standardized delivery of CBT-I core modules at low cost [85,86], and emerging technologies (predominantly virtual reality) may reduce presleep somatic arousal through immersive relaxation [87,88], although their adoption is constrained by hardware accessibility and user comfort [84].

Country income level emerged as an effect modifier for insomnia severity, with high-income countries showing substantially larger improvements than upper-middle-income countries. Several contextual factors may explain this disparity. First, high-income countries generally possess more mature digital health infrastructure, including higher smartphone penetration, more stable internet connectivity [89], and established pathways for digital therapeutic reimbursement in several leading high-income countries [90]. Second, the digital interventions tested in high-income settings were more likely to be culturally adapted and linguistically aligned with the target population, whereas those applied in upper-middle-income countries often involved direct translations of Western-developed programs without substantive cultural tailoring, potentially reducing engagement and efficacy [91]. Third, differences in health care system integration, including the availability of primary care referrals to digital interventions, may influence adherence and clinical outcomes [92]. This finding underscores the urgent need for implementation research that addresses the digital divide in insomnia care, particularly through the development of locally adapted, affordable, and scalable digital solutions for low- and middle-income settings [93].

Comparator type showed a significant interaction for total sleep time, with passive comparators yielding larger effect sizes than low-intensity active controls. This pattern is methodologically expected: low-intensity active controls, such as sleep hygiene education or brief counseling, confer nonnegligible therapeutic benefits themselves [94], thereby reducing the between-group difference [95]. This finding suggests that the incremental benefit of DHIs over minimal active interventions, while statistically significant, may be more modest than suggested by comparisons against waiting list or treatment-as-usual controls. Clinicians and policymakers should therefore interpret effect sizes from passive-controlled trials with caution, recognizing that the real-world added value of DHIs relative to existing low-intensity services may be smaller than trial estimates suggest [95,96].

However, several considerations temper the interpretation of these statistically significant findings. In terms of clinical translation, statistical significance does not equate to clinical importance. Although effect sizes for insomnia severity and sleep quality were moderate to large, conversion to original scale scores revealed changes that only approached or fell short of previously recommended minimal clinically important difference thresholds [77-79]. For total sleep time, the improvement translated to only a few minutes, well below the threshold generally considered clinically meaningful [81]. This indicates that although current evidence supports the average effectiveness of DHIs at the population level, the actual magnitude of benefit for individual patients remains uncertain. Furthermore, the 95% PIs were wide for all primary outcomes, and those for insomnia severity, sleep quality, and several secondary outcomes (eg, sleep onset latency and wake after sleep onset) encompassed the null value. This suggests that, in future real-world clinical settings, individual patient responses to DHIs may range from substantial benefit to little or no effect. In addition, GRADE assessments indicated low-to-moderate certainty for primary outcomes, with downgrading due to high heterogeneity, risk of small-study effects for some outcomes, and concerns about outcome measurement bias in most studies because participants were aware of their group assignment. Therefore, the present findings should be interpreted as exploratory rather than definitive [97].

Compared with previous meta-analyses, our effect estimates are broadly consistent with those of earlier reviews focusing on digital CBT-I [98]. Beyond this confirmation, our review offers 3 additional findings worthy of further discussion. First, whereas prior reviews typically combined different digital intervention formats [99], our subgroup analyses revealed differential effects across intervention modalities. For instance, remote interaction showed larger effect sizes than autonomous self-management for sleep efficiency, suggesting that regular therapist supervision, personalized feedback, and adherence reinforcement may be key components that amplify treatment effects [100]. Second, we reported PIs, which previous reviews have infrequently reported. This approach explicitly highlights the substantial uncertainty in individual-level treatment responses. It also provides a more cautious evidentiary framework for clinical translation [38,39]. Third, through conversion of pooled effect sizes to original scale scores, we identified a gap between statistically significant effects and clinically meaningful differences [83]. However, such conversions depend on the representativeness of the SDs used across studies, and therefore have limited precision [101]. This observation should be interpreted cautiously [83]. Future studies that report the proportion of participants achieving a minimal clinically important difference may provide a more clinically useful metric than between-group mean differences alone [102,103].

We observed a nonsignificant trend toward larger subjective than objective improvements, a pattern that aligns with the work of Ahn et al [96]. However, the subgroup differences were not statistically significant, so this pattern should be interpreted cautiously rather than as definitive evidence of a subjective-objective discrepancy. This discrepancy can be interpreted from 2 perspectives. First, from a pathophysiological standpoint, patients with insomnia may experience cortical microarousals during polysomnographically defined sleep, leading to a subjective experience of wakefulness, a dissociation between cortical arousal and subjective perception [84,104]. This phenomenon is consistently documented in paradoxical insomnia, where subjective sleep reports markedly underestimate objective sleep duration [105]. Prior evidence suggests that DHIs, through psychoeducation, cognitive restructuring, and relaxation training, can effectively reduce sleep-related anxiety and catastrophic interpretations of nocturnal microarousals, thereby preferentially narrowing the gap between perceived and objective sleep [91,106] rather than directly altering physiological sleep architecture. This observed pattern therefore provides insight into the potential mechanisms of DHIs: they may operate through cognitive, behavioral, and physiological pathways to improve subjective sleep experience. Second, from a methodological standpoint, the subjective-objective discrepancy may partly reflect study design biases. Given the inherent difficulty of blinding participants and researchers in digital intervention studies, and given that primary outcomes were patient-reported, awareness of receiving an active intervention may systematically inflate subjective ratings through expectancy and placebo effects [76,107].

These subgroup observations must be interpreted with statistical and evidentiary caution. The observed gradient in insomnia severity across intervention categories did not reach statistical significance under the random-effects model, and the ranking may reflect imbalances in study numbers across subgroups, baseline characteristics, comparator types, or other confounders rather than true differences in treatment efficacy [38,39,108]. Moreover, these subgroup comparisons are based on indirect between-study comparisons rather than direct head-to-head RCTs; the emerging-technology subgroup consisted of only a few small studies for several outcomes, limiting the stability of its pooled estimates [109]. Therefore, these findings should be regarded as hypothesis-generating and exploratory, and the relative superiority of different modalities in specific populations should be tested in adequately powered, head-to-head RCTs [109]. Clinicians may consider these findings as reference information for individualized decision-making, but they do not yet provide sufficient evidence to support standard treatment recommendations [110].

### Strengths and Limitations

This review followed established methodological standards for systematic reviews and meta-analyses, with comprehensive literature searches across 11 databases and multiple gray literature sources. The statistical approach incorporated HKSJ-corrected random-effects models, PIs, and GRADE assessments, which together strengthened the reliability of the findings [111]. The inclusion of a large number of randomized trials provided a substantial evidence base for quantitative synthesis. Compared with prior reviews that often focused on single intervention formats or limited outcome domains, this review examined 3 intervention modalities simultaneously and evaluated a broad range of sleep-related outcomes. Subgroup analyses were prespecified in the study protocol. They explored potential sources of heterogeneity across intervention types, comparator conditions, diagnostic criteria, and country income levels. These features allow for a more differentiated interpretation of the evidence than previous syntheses have provided.

This review has several limitations. The *I*² values for several primary outcomes were high. Although this reflects a large proportion of observed variation attributable to between-study differences, *I*² is a relative measure of inconsistency rather than an absolute measure of heterogeneity [39]. The *τ* values and PIs reported alongside each estimate therefore provide a more clinically informative assessment of heterogeneity in this review. The high *I*² values may be partly attributable to differences in intervention types, comparator settings, and diagnostic criteria, and may also reflect the relatively high precision with which some study-level estimates were measured. Furthermore, the overall pooled estimates presented in this review should be interpreted as descriptive summaries of the average effects across the included DHI types. Given the clinical and methodological diversity across interventions, these estimates do not represent a stable or uniform effect that can be generalized to all digital insomnia interventions. Most studies were at risk of bias because participants were aware of their group assignment, which may have inflated the effects on subjective outcomes [107]. GRADE assessments indicated very low-to-moderate certainty for primary outcomes, and the 95% PIs suggested that clinical responses could range from substantial benefit to no effect, necessitating cautious interpretation [38,39,111]. Small-study effects were suggested by Egger tests for several outcomes, although trim-and-fill adjustments did not materially alter the pooled estimates; these findings may reflect publication bias but can also arise from other causes [112]. Some subgroup analyses included only 1 to 3 small studies, and subgroup comparisons were based on indirect evidence rather than head-to-head RCTs; all subgroup findings should therefore be considered hypothesis-generating rather than clinically definitive [109]. We acknowledge that network meta-analysis was not performed because the heterogeneity across intervention categories and the poorly connected evidence network precluded the transitivity assumption [113]. Component network meta-analysis was also not feasible due to insufficiently standardized component-level reporting in the included studies [114].

Several further limitations concern the evidence base and its generalizability. Most studies assessed only immediate postintervention effects (8 to 12 weeks) and lacked long-term follow-up (≥6 months); given that insomnia is a chronic relapsing condition, the durability of treatment effects remains unknown [115,116]. There is also insufficient reporting of null or nonsignificant results, which may have inflated the pooled effect estimates [112]. The majority of studies used passive comparators, and head-to-head comparisons between different DHI modalities are lacking, limiting our ability to determine their optimal positioning in the treatment algorithm [117]. The evidence base is heavily concentrated in high-income countries and in populations of young-to-middle-aged, highly educated, urban residents, severely limiting generalizability to older adults, lower-education groups, and low-income populations—a digital divide. Older adults frequently face barriers such as technology anxiety, age-related sensory decline, and unfriendly user interfaces [118]; lower-education groups may struggle to understand abstract psychoeducational materials and behavioral instructions [119]; and low-income populations often face not only affordability constraints regarding smartphones and data plans [120] but also disadvantaged sleep environments, higher life stress, and greater comorbidity burdens [121], all of which independently worsen insomnia and reduce the applicability of standardized interventions [122]. Furthermore, assessments of small-study effects, including funnel plot visual inspection and Egger tests, should be interpreted with caution, as several meta-analyses included relatively small numbers of studies, which limits the statistical power of these detection methods [123].

Future research should therefore (1) prioritize studies targeting diverse populations and exploring adaptive strategies such as simplified interfaces, voice interaction, community support, and hybrid models [118,124]; (2) conduct hybrid model RCTs in community health centers [125]; (3) perform cognitive interviews with low-health-literacy groups to optimize intervention content [126]; and (4) clarify whether digital interventions should serve as a complement rather than a substitute for traditional in-person care in resource-limited settings [127]. In addition, the field would benefit from consensus on core outcome sets and standardized intervention reporting, the development of active comparator designs and digital placebos, head-to-head comparisons of different DHI modalities, extended follow-up periods (≥12 months), encouraging publication of null results, and exploration of artificial intelligence–driven adaptive intervention systems to move digital sleep care from standardized treatment toward precision dynamic management [128].

### Conclusions

This systematic review and meta-analysis of 44 RCTs evaluated the efficacy of DHIs for insomnia and compared, by intervention functional category, the effects of 3 digital intervention modalities—autonomous self-management, remote interaction, and emerging technologies. The evidence suggests that DHIs may improve insomnia severity, sleep efficiency, sleep quality, and several related symptoms, yet considerable uncertainty remains regarding their translation into clinically meaningful benefits, with marked interindividual variability.

Subgroup analyses revealed several notable patterns that warrant emphasis. First, country income level emerged as an effect modifier for insomnia severity, with high-income countries demonstrating substantially larger effects than upper-middle-income countries—a finding that underscores the pervasive digital divide in sleep medicine. Second, intervention functional category modified sleep efficiency outcomes, with remote interaction and autonomous self-management showing comparable effects, both larger than emerging technologies. Third, comparator type influenced total sleep time, with passive comparators yielding larger effects than low-intensity active controls, highlighting the importance of control group selection in trial interpretation. However, the observed gradient across intervention modalities should be considered exploratory and hypothesis-generating at this stage, rather than a basis for clinical triage recommendations. The wide PIs, low-to-moderate GRADE certainty, and concentration of evidence among young-to-middle-aged adults in high-income countries collectively limit the translation of these findings into individualized clinical decision-making. The improvement in subjective sleep experience showed a nonsignificant trend toward being larger than the change in objective sleep duration, suggesting that the core value of DHIs may lie in improving sleep perception and subjective experience rather than substantially increasing objective sleep time.

In conclusion, DHIs may serve as a low-intensity, highly accessible initial option in a stepped-care model for insomnia, particularly for patients with mild-to-moderate insomnia, those with self-motivation, and those who cannot readily access face-to-face CBT-I. However, given the low-to-moderate certainty of the current evidence and the clear digital divide, these suggestions should be applied with clinical judgment, carefully weighing individual patient characteristics, digital accessibility, and treatment uncertainty. This study provides exploratory evidence to inform the initial clinical application of DHIs in insomnia management, but future head-to-head comparative and implementation science studies in diverse populations are needed to advance the precise and equitable use of DHIs.

## Supplementary material

10.2196/90905 Multimedia Appendix 1 Search strategies for all databases.

10.2196/90905 Multimedia Appendix 2 Related supplementary figures.

10.2196/90905 Multimedia Appendix 3 The trial registration numbers and registration platforms for each included study.

10.2196/90905 Checklist 1 PRISMA 2020 abstract checklist.

10.2196/90905 Checklist 2 PRISMA 2020 expanded checklist.

10.2196/90905 Checklist 3 PRISMA-S checklist.

## Abbreviations

CBT-I: cognitive behavioral therapy for insomnia
DHI: digital health intervention
GRADE: Grading of Recommendations Assessment, Development and Evaluation
HKSJ: Hartung-Knapp-Sidik-Jonkman correction
ISI: Insomnia Severity Index
ITT: intention-to-treat
PI: prediction interval
PRISMA: Preferred Reporting Items for Systematic Reviews and Meta-Analyses
PRISMA-S: Preferred Reporting Items for Systematic Reviews and Meta-Analyses literature search extension
PROSPERO: International Prospective Register of Systematic Reviews
PSQI: Pittsburgh Sleep Quality Index
RCT: randomized controlled trial
RoB 2: Cochrane Risk of Bias 2.0 tool
SMD: standardized mean difference
WHO: World Health Organization
