# Supplementary material for: Efficacy of Digital Health Interventions for Insomnia: Systematic Review and Meta-Analysis of Randomized Controlled Trials
Source: J Med Internet Res. 2026 Nov 13;28:e90905. doi: 10.2196/90905 (PMC13766658; doi:10.2196/90905)
Supplement: Supplementary file 1 [file jmir-v28-e90905-s001.docx]

**Multimedia Appendix 1**

**Methods 1 –** Database search strategy.

**1.Pub Med**

| **ID** | **Search string** | **Results** |
| --- | --- | --- |
| 1 | (((((((((((((((((((("Digital Health"[Mesh]) OR ("Telemedicine"[Mesh])) OR ("Wearable Electronic Devices"[Mesh])) OR ("Mobile Applications"[Mesh])) OR ("Smartphone"[Mesh])) OR ("Cell Phone"[Mesh])) OR ("Web Browser"[Mesh])) OR ("Online Systems"[Mesh])) OR ("Internet"[Mesh])) OR ("Computers"[Mesh])) OR ("Software"[Mesh])) OR ("Virtual Reality"[Mesh])) OR ("Augmented Reality"[Mesh])) OR ("Electronic Health Records"[Mesh])) OR ("Artificial Intelligence"[Mesh])) OR ("Machine Learning"[Mesh])) OR ("Big Data"[Mesh])) OR ("Precision Medicine"[Mesh])) OR ("Social Media"[Mesh])) OR ("Remote Patient Monitoring"[Mesh])) OR (((((((((((((((((((((((((((((((((((((((((((((((((((digital[Title/Abstract]) OR (Digital Health Technolog*[Title/Abstract])) OR (digital health intervention[Title/Abstract])) OR (electronic-health[Title/Abstract])) OR (e-health[Title/Abstract])) OR (e intervention[Title/Abstract])) OR (Virtual Medicine[Title/Abstract])) OR (Tele Referral[Title/Abstract])) OR (Mobile Health[Title/Abstract])) OR (mHealth[Title/Abstract])) OR (Telehealth[Title/Abstract])) OR (eHealth[Title/Abstract])) OR (Telecare[Title/Abstract])) OR (Tele Care[Title/Abstract])) OR (Wearable Electronic Device[Title/Abstract])) OR (Wearable Device[Title/Abstract])) OR (Wearable Technolog*[Title/Abstract])) OR (wearable[Title/Abstract])) OR (Mobile Application[Title/Abstract])) OR (Mobile App[Title/Abstract])) OR (Portable Electronic App*[Title/Abstract])) OR (Portable Software App*[Title/Abstract])) OR (Smartphone App[Title/Abstract])) OR (App[Title/Abstract])) OR (application[Title/Abstract])) OR (Cellular Phone[Title/Abstract])) OR (Cellular Telephone[Title/Abstract])) OR (Cell Phones[Title/Abstract])) OR (Mobile Phone[Title/Abstract])) OR (Mobile Telephone[Title/Abstract])) OR (Portable Cellular Phone[Title/Abstract])) OR (Transportable Cellular Phone[Title/Abstract])) OR (web[Title/Abstract])) OR (web-based[Title/Abstract])) OR (Online[Title/Abstract])) OR (VR[Title/Abstract])) OR (Mixed Realit*[Title/Abstract])) OR (AR[Title/Abstract])) OR (health information technology[Title/Abstract])) OR (EHR[Title/Abstract])) OR (AI (Artificial Intelligence[Title/Abstract]))) OR (personalized medicine[Title/Abstract])) OR (Social Medium[Title/Abstract])) OR (Twitter Messaging[Title/Abstract])) OR (Mobile Social Network[Title/Abstract])) OR (Mobile Social Media[Title/Abstract])) OR (wechat[Title/Abstract])) OR (tele-consultation[Title/Abstract])) OR (video consultation[Title/Abstract])) OR (telemonitoring[Title/Abstract])) OR (e-reader[Title/Abstract])) | 2,921,118 |
| 2 | ((((((((((((((((((((((DIMS (Disorders of Initiating[Title/Abstract] AND Maintaining Sleep[Title/Abstract])) OR (Disorders of Initiating[Title/Abstract] AND Maintaining Sleep[Title/Abstract])) OR (Sleeplessness[Title/Abstract])) OR (Insomnia Disorder[Title/Abstract])) OR (Insomnia*[Title/Abstract])) OR (Chronic Insomnia[Title/Abstract])) OR (Early Awakening[Title/Abstract])) OR (Nonorganic Insomnia[Title/Abstract])) OR (Primary Insomnia[Title/Abstract])) OR (Psychophysiological Insomnia[Title/Abstract])) OR (Rebound Insomnia[Title/Abstract])) OR (Secondary Insomnia[Title/Abstract])) OR (Sleep Initiation Dysfunction[Title/Abstract])) OR (Transient Insomnia[Title/Abstract])) OR (Sleep Wake Disorder[Title/Abstract])) OR (Sleep Disorder[Title/Abstract])) OR (Short Sleeper Syndrome[Title/Abstract])) OR (Short Sleep Phenotype[Title/Abstract]))) OR (sleep difficult*[Title/Abstract])) OR (sleep disturbance*[Title/Abstract])) OR (sleep wake disorder*[Title/Abstract])) OR (dyssomnia*[Title/Abstract]) | 72,036 |
| 3 | (((("Randomized Controlled Trial" [Publication Type]) OR ("Controlled Clinical Trial" [Publication Type])) OR (randomized clinical trial)) OR (RCT)) OR (CCT) | 1,004,632 |
| 4 | #1 AND #2 AND #3 | 1,150 |

**2.CNKI [In Chinese]**

| **ID** | **Search string** | **Search string (English translation for reference)** | **Results** |
| --- | --- | --- | --- |
| 1 | 主题=（智能 + 应用程序 + 软件 + 网络 + 数字 + 在线 + 虚拟现实 + 增强现实 + 可穿戴 + 计算机 + 微信 + 电子 + 移动健康 + 远程医疗 + 远程健康） | **Topic =** (intelligent + application + software + network + digital + online + virtual reality + augmented reality + wearable + computer + WeChat + electronic + mobile health + telemedicine + telehealth) | 995.58w |
| 2 | 主题=（睡眠 + 失眠 + 入睡困难） | **Topic =** (sleep + insomnia + difficulty falling asleep) | 18.95w |
| 3 | 主题=（RCT + 随机对照 + CCT + 类实验） | **Topic =** (RCT + randomized controlled trial + CCT + controlled clinical trial + quasi-experimental) | 8.57w |
| 4 | #1 + #2 + #3 | #1 + #2 + #3 | 128 |

***The original search strings were executed in Chinese databases (CNKI). The English translations are provided for reference only and do not represent the exact search syntax used.***

**3.Cochrane library**

| **ID** | **Search string** | **Results** |
| --- | --- | --- |
| #1 | MeSH descriptor: [Digital Health] explode all trees | 249 |
| #2 | Digital Health OR Digital Health Technology OR digital health intervention OR electronic-health OR e-health OR e intervention OR mobile health OR m-health OR telemedicine OR telehealth OR Wearable Electronic Devices OR wearable OR wearable device OR mobile application OR smartphone OR app OR mobile phone OR web OR web-based OR online OR internet OR internet intervention OR computer OR software OR digital OR virtual reality OR augmented reality OR VR OR AR OR health information technology OR electronic health record OR EHR OR artificial intelligence OR AI OR machine learning OR big data OR personalized medicine OR precision medicine OR social media OR wechat OR tele-consultation OR video consultation OR remote monitoring OR telemonitoring OR application OR e-reader | 459,824 |
| #3 | #1 OR #2 | 459,824 |
| #4 | MeSH descriptor: [Sleep Initiation and Maintenance Disorders] explode all trees | 4,506 |
| #5 | MeSH descriptor: [Dyssomnias] explode all trees | 11,340 |
| #6 | Insomnia OR Sleep Wake Disorders OR sleeplessness OR sleep initiation and maintenance disorders OR disorders of initiating and maintaining sleep OR DIMS OR chronic insomnia OR primary insomnia OR psychophysiological insomnia OR nonorganic insomnia OR transient insomnia OR early awakening OR sleep initiation dysfunction OR sleep difficult* OR sleep disturbance* OR sleep wake disorder* | 32,037 |
| #7 | #4 OR #5 OR #6 | 37,601 |
| #8 | MeSH descriptor: [Randomized Controlled Trial] explode all trees | 34 |
| #9 | MeSH descriptor: [Controlled Clinical Trial] explode all trees | 37 |
| #10 | randomized clinical trial OR RCT OR CCT | 1,019,831 |
| #11 | #8 OR #9 OR #10 | 1,019,859 |
| #12 | #3 AND #7 AND #11 | 7,110 |

**4.Web of Science**

| **ID** | **Search string** | **Results** |
| --- | --- | --- |
| #1 | **TS=(Digital Health OR Digital Health Technology OR digital health intervention OR electronic-health OR e-health OR e intervention OR mobile health OR m-health OR telemedicine OR telehealth OR Wearable Electronic Devices OR wearable OR wearable device OR mobile application OR smartphone OR app OR mobile phone OR web OR web-based OR online OR internet OR internet intervention OR computer OR software OR digital OR virtual reality OR augmented reality OR VR OR AR OR health information technology OR electronic health record OR EHR OR artificial intelligence OR AI OR machine learning OR big data OR personalized medicine OR precision medicine OR social media OR wechat OR tele-consultation OR video consultation OR remote monitoring OR telemonitoring OR application OR e-reader)** | 12,355,482 |
| #2 | TS=(Sleep Initiation and Maintenance Disorders OR Insomnia OR Sleep Wake Disorders OR insomnia* OR sleeplessness OR disorders of initiating and maintaining sleep OR DIMS OR chronic insomnia OR primary insomnia OR psychophysiological insomnia OR nonorganic insomnia OR transient insomnia OR early awakening OR sleep initiation dysfunction OR sleep difficult* OR sleep disturbance* OR sleep wake disorder* OR dyssomnia*) | 142,496 |
| #3 | TS=(**Randomized Controlled Trial OR Controlled Clinical Trial OR randomized clinical trial OR RCT OR CCT**) | 988,679 |
| #4 | #1 AND #2 AND #3 | 3,053 |

**5.Scopus**

| **ID** | **Search string** | **Results** |
| --- | --- | --- |
| #1 | ( TITLE-ABS-KEY ( Digital Health ) OR TITLE-ABS-KEY ( Digital Health Technology ) OR TITLE-ABS-KEY ( digital health intervention ) OR TITLE-ABS-KEY ( electronic-health ) OR TITLE-ABS-KEY ( e-health ) OR TITLE-ABS-KEY ( e intervention ) OR TITLE-ABS-KEY ( mobile health ) OR TITLE-ABS-KEY ( m-health ) OR TITLE-ABS-KEY ( telemedicine ) OR TITLE-ABS-KEY ( telehealth ) OR TITLE-ABS-KEY ( Wearable Electronic Devices ) OR TITLE-ABS-KEY ( wearable ) OR TITLE-ABS-KEY ( wearable device ) OR TITLE-ABS-KEY ( mobile application ) OR TITLE-ABS-KEY ( smartphone ) OR TITLE-ABS-KEY ( app ) OR TITLE-ABS-KEY ( mobile phone ) OR TITLE-ABS-KEY ( web ) OR TITLE-ABS-KEY ( web-based ) OR TITLE-ABS-KEY ( online ) OR TITLE-ABS-KEY ( internet ) OR TITLE-ABS-KEY ( internet intervention ) OR TITLE-ABS-KEY ( computer ) OR TITLE-ABS-KEY ( software ) OR TITLE-ABS-KEY ( digital ) OR TITLE-ABS-KEY ( virtual reality ) OR TITLE-ABS-KEY ( augmented reality ) OR TITLE-ABS-KEY ( VR ) OR TITLE-ABS-KEY ( AR ) OR TITLE-ABS-KEY ( health information technology ) OR TITLE-ABS-KEY ( electronic health record ) OR TITLE-ABS-KEY ( EHR ) OR TITLE-ABS-KEY ( artificial intelligence ) OR TITLE-ABS-KEY ( AI ) OR TITLE-ABS-KEY ( machine learning ) OR TITLE-ABS-KEY ( big data ) OR TITLE-ABS-KEY ( personalized medicine ) OR TITLE-ABS-KEY ( precision medicine ) OR TITLE-ABS-KEY ( social media ) OR TITLE-ABS-KEY ( wechat ) OR TITLE-ABS-KEY ( tele-consultation ) OR TITLE-ABS-KEY ( video consultation ) OR TITLE-ABS-KEY ( remote monitoring ) OR TITLE-ABS-KEY ( telemonitoring ) OR TITLE-ABS-KEY ( application ) OR TITLE-ABS-KEY ( e-reader) ) | 21,646,584 |
| #2 | ( TITLE-ABS-KEY ( Sleep Initiation and Maintenance Disorders ) OR TITLE-ABS-KEY ( Insomnia ) OR TITLE-ABS-KEY ( Sleep Wake Disorders ) OR TITLE-ABS-KEY ( sleeplessness ) OR TITLE-ABS-KEY ( disorders of initiating and maintaining sleep ) OR TITLE-ABS-KEY ( DIMS ) OR TITLE-ABS-KEY ( chronic insomnia ) OR TITLE-ABS-KEY ( primary insomnia ) OR TITLE-ABS-KEY ( psychophysiological insomnia ) OR TITLE-ABS-KEY ( nonorganic insomnia ) OR TITLE-ABS-KEY ( transient insomnia ) OR TITLE-ABS-KEY ( early awakening ) OR TITLE-ABS-KEY ( sleep initiation dysfunction ) OR TITLE-ABS-KEY ( sleep difficult* ) OR TITLE-ABS-KEY ( sleep disturbance* ) OR TITLE-ABS-KEY ( sleep wake disorder* ) OR TITLE-ABS-KEY ( dyssomnia*) ) | 209,106 |
| #3 | ( TITLE-ABS-KEY ( Randomized Controlled Trial ) OR TITLE-ABS-KEY ( Controlled Clinical Trial ) OR TITLE-ABS-KEY ( randomized clinical trial ) OR TITLE-ABS-KEY ( RCT ) OR TITLE-ABS-KEY ( CCT ) ) | 1,846,564 |
| #4 | #1 AND #2 AND #3 | 5,863 |

**6.Wanfang [In Chinese]**

| **ID** | **Search string** | **Search string (English translation for reference)** | **Results** |
| --- | --- | --- | --- |
| 1 | 主题=（智能 OR 应用程序 OR 软件 OR 网络 OR 数字 OR 在线 OR 虚拟现实 OR 增强现实 OR 可穿戴 OR 计算机 OR 微信 OR 电子 OR 移动健康 OR 远程） | **Topic =** (intelligent OR application OR software OR network OR digital OR online OR virtual reality OR augmented reality OR wearable OR computer OR WeChat OR electronic OR mobile health OR telehealth) | 8,597,486 |
| 2 | 主题=（睡眠 OR 失眠 OR 入睡困难） | **Topic =** (sleep OR insomnia OR difficulty falling asleep) | 234,060 |
| 3 | 主题=（RCT OR 随机对照 OR CCT OR 类实验 OR 准实验 OR 非随机对照 OR 准随机） | **Topic =** (RCT OR randomized controlled trial OR CCT OR controlled clinical trial OR quasi-experimental OR non-randomized controlled trial OR quasi-randomized) | 3,194,669 |
| 4 | #1 AND #2 AND #3 | #1 AND #2 AND #3 | 4,267 |

***The original search strings were executed in Chinese databases (Wanfang). The English translations are provided for reference only and do not represent the exact search syntax used.***

**7.VIP [In Chinese]**

| **ID** | **Search string** | **Search string (English translation for reference)** | **Results** |
| --- | --- | --- | --- |
| 1 | 主题=（智能 OR 应用程序 OR 软件 OR 网络 OR 数字 OR 在线 OR 虚拟现实 OR 增强现实 OR 可穿戴 OR 计算机 OR 微信 OR 电子 OR 移动健康 OR 远程） | **Topic =** (intelligent OR application OR software OR network OR digital OR online OR virtual reality OR augmented reality OR wearable OR computer OR WeChat OR electronic OR mobile health OR telemedicine) | 4,786,700 |
| 2 | 主题=（睡眠 OR 失眠 OR 入睡困难） | **Topic =** (sleep OR insomnia OR difficulty falling asleep) | 116,163 |
| 3 | 题名或关键词=（RCT OR 随机对照 OR CCT OR 类实验 OR 准实验 OR 非随机对照 OR 准随机） | **Title/Keyword =** (RCT OR randomized controlled trial OR CCT OR controlled clinical trial OR quasi-experimental OR non-randomized controlled trial OR quasi-randomized) | 38,412 |
| 4 | #1 AND #2 AND #3 | #1 AND #2 AND #3 | 225 |

***The original search strings were executed in Chinese databases (VIP). The English translations are provided for reference only and do not represent the exact search syntax used.***

**8.CBM [In Chinese]**

| **ID** | **Search string** | **Search string (English translation for reference)** | **Results** |
| --- | --- | --- | --- |
| 1 | "人工智能"[不加权:扩展] | "artificial intelligence" [unweighted: expanded] | 45,085 |
| 2 | "社交网络"[不加权:扩展] | "social network" [unweighted: expanded] | 1,388 |
| 3 | "数字技术"[不加权:扩展] OR "计算机"[不加权:扩展] | "digital technology" [unweighted: expanded] OR "computer" [unweighted: expanded] | 28,770 |
| 4 | "虚拟现实"[不加权:扩展] | "virtual reality" [unweighted: expanded] | 2,877 |
| 5 | "增强现实"[不加权:扩展] | "augmented reality" [unweighted: expanded] | 727 |
| 6 | "可穿戴电子设备"[不加权:扩展] | "wearable electronic devices" [unweighted: expanded] | 1,474 |
| 7 | "电子"[不加权:扩展] | "electronic" [unweighted: expanded] | 76 |
| 8 | "远程护理"[不加权:扩展] OR "教育, 远程"[不加权:扩展] OR "远程医学"[不加权:扩展] OR "远程康复"[不加权:扩展] OR "远程咨询"[不加权:扩展] OR "机器人"[不加权:扩展] | "tele-nursing" [unweighted: expanded] OR "education, distance" [unweighted: expanded] OR "telemedicine" [unweighted: expanded] OR "telerehabilitation" [unweighted: expanded] OR "tele-counseling" [unweighted: expanded] OR "robot" [unweighted: expanded] | 18,109 |
| 9 | "互联网干预"[不加权:扩展] | "internet intervention" [unweighted: expanded] | 71 |
| 10 | "移动应用"[不加权:扩展] OR "便携式电话"[不加权:扩展] OR "手机使用"[不加权:扩展] | "mobile application" [unweighted: expanded] OR "portable phone" [unweighted: expanded] OR "mobile phone use" [unweighted: expanded] | 4,647 |
| 11 | "微信"[常用字段:智能] | "WeChat" [common field: smart] | 14,905 |
| 12 | "软件"[不加权:扩展] | "software" [unweighted: expanded] | 43,841 |
| 13 | (#12) OR (#11) OR (#10) OR (#9) OR (#8) OR (#7) OR (#6) OR (#5) OR (#4) OR (#3) OR (#2) OR (#1) | (#12) OR (#11) OR (#10) OR (#9) OR (#8) OR (#7) OR (#6) OR (#5) OR (#4) OR (#3) OR (#2) OR (#1) | 145,582 |
| 14 | "睡眠"[不加权:扩展] OR "睡眠障碍"[不加权:扩展] OR "入睡和睡眠障碍"[不加权:扩展] OR "失眠症"[不加权:扩展] | "sleep" [unweighted: expanded] OR "sleep disorder" [unweighted: expanded] OR "disorders of initiating and maintaining sleep" [unweighted: expanded] OR "insomnia" [unweighted: expanded] | 95,290 |
| 15 | "随机对照试验"[不加权:扩展] | "randomized controlled trial" [unweighted: expanded] | 200,395 |
| 16 | "类实验"[常用字段:智能] OR "准实验"[常用字段:智能] OR "非随机对照"[常用字段:智能] | "quasi-experimental" [common field: smart] OR "quasi-experiment" [common field: smart] OR "non-randomized controlled" [common field: smart] | 3,947 |
| 17 | (#15) OR (#16) | (#15) OR (#16) | 203,675 |
| 18 | (#13) AND (#14) AND (#17) | (#13) AND (#14) AND (#17) | 38 |

***The original search strings were executed in Chinese databases (CBM). The English translations are provided for reference only and do not represent the exact search syntax used.***

**9.AASM**

| AASM | 2026.7.18 | Manual retrieval of subject classification | 268 |
| --- | --- | --- | --- |

**10.CINAHL**

| **ID** | **Search string** | **Results** |
| --- | --- | --- |
| 1 | SU (MH "Digital Health+/NU" OR MH "Digital Technology" OR digital health intervention OR MH "Electronic Health Records" OR e-health OR e intervention OR MH "Smartphone" OR MH "Mobile Applications" OR MH "Digital Media" OR MH "Telephone" OR MH "Cellular Phone" OR app OR e-reader OR application OR m-health OR MH "Software" OR MH "Web Browsers" OR web OR MH "Online Services" OR MH "Virtual Reality" OR MH "Augmented Reality" OR VR OR AR OR MH "Internet-Based Intervention" OR MH "Wearable Sensors" OR MH "Smart Glasses" OR MH "Computer Systems" OR wechat OR MH "Telemedicine" OR MH "Telehealth" OR mobile health OR MH "Artificial Intelligence") | 276,768 |
| 2 | SU (MH "Insomnia" OR MH "Sleep Disorders" OR Insomnia Disorder OR Chronic Insomnia OR DIMS sleep problem) | 25,734 |
| 3 | SU (MH "Randomized Controlled Trials" OR MH "Nonrandomized Trials" OR MH "Clinical Trials" OR randomized clinical trial OR RCT OR CCT) | 341,507 |
| 4 | #1 AND #2 AND #3 | 104 |

**11.Embase**

| **ID** | **Search string** | **Results** |
| --- | --- | --- |
| #1 | 'digital health'/exp OR 'digital health' | 39,124 |
| #2 | 'digital health technology'/exp OR 'digital health technology' OR 'digital health intervention':ti,ab,kw OR 'electronic health':ti,ab,kw OR 'telehealth':ti,ab,kw OR 'mobile health':ti,ab,kw OR 'telemedicine':ti,ab,kw OR 'wearable electronic device':ti,ab,kw OR 'wearable device':ti,ab,kw OR 'mobile application':ti,ab,kw OR 'smartphone':ti,ab,kw OR app:ti,ab,kw OR 'mobile phone':ti,ab,kw OR 'web':ti,ab,kw OR 'web-based intervention':ti,ab,kw OR 'online':ti,ab,kw OR 'internet':ti,ab,kw OR 'health information technology':ti,ab,kw OR 'computer':ti,ab,kw OR 'software':ti,ab,kw OR 'digital':ti,ab,kw OR 'virtual reality':ti,ab,kw OR 'augmented reality':ti,ab,kw OR 'electronic health record':ti,ab,kw OR 'artificial intelligence':ti,ab,kw OR 'machine learning':ti,ab,kw OR 'big data':ti,ab,kw OR 'personalized medicine':ti,ab,kw OR 'precision medicine':ti,ab,kw OR 'social media':ti,ab,kw OR 'wechat':ti,ab,kw OR 'tele-consultation':ti,ab,kw OR 'video consultation':ti,ab,kw OR 'remote monitoring':ti,ab,kw OR 'telemonitoring':ti,ab,kw OR 'application':ti,ab,kw OR 'e-reader':ti,ab,kw | 3,939,488 |
| #3 | #1 OR #2 | 3,947,279 |
| #4 | 'insomnia'/exp | 112,418 |
| #5 | 'dyssomnia'/exp | 266,247 |
| #6 | #4 OR #5 | 266,247 |
| #7 | 'Sleep Initiation and Maintenance Disorders':ti,ab,kw OR 'Insomnia':ti,ab,kw OR 'Sleep Wake Disorders':ti,ab,kw OR 'insomnia*':ti,ab,kw OR 'sleeplessness':ti,ab,kw OR 'disorders of initiating and maintaining sleep':ti,ab,kw OR 'chronic insomnia':ti,ab,kw OR 'primary insomnia':ti,ab,kw OR 'psychophysiological insomnia':ti,ab,kw OR 'nonorganic insomnia':ti,ab,kw OR 'transient insomnia':ti,ab,kw OR 'early awakening':ti,ab,kw OR 'sleep initiation dysfunction':ti,ab,kw OR 'sleep difficult*':ti,ab,kw OR 'sleep disturbance*':ti,ab,kw OR 'sleep wake disorder*':ti,ab,kw OR 'dyssomnia*':ti,ab,kw | 114,396 |
| #8 | #6 OR #7 | 313,879 |
| #9 | 'randomized controlled trial'/exp | 1,224,695 |
| #10 | 'controlled clinical trial'/exp | 1,416,496 |
| #11 | 'randomized clinical trial':ti,ab,kw OR rct:ti,ab,kw OR cct:ti,ab,kw | 183,721 |
| #12 | #9 OR #10 OR #11 | 1,484,561 |
| #13 | #3 AND #8 AND #12 | 4,321 |

To avoid overlooking relevant literature, we checked the reference lists of systematic reviews published in the last two years and performed manual searches to identify additional relevant studies.
