# Supplementary material for: Efficacy of Digital Health Interventions for Insomnia: Systematic Review and Meta-Analysis of Randomized Controlled Trials
Source: J Med Internet Res. 2026 Nov 13;28:e90905. doi: 10.2196/90905 (PMC13766658; doi:10.2196/90905)
Supplement: Supplementary file 2 [file jmir-v28-e90905-s002.docx]

**Multimedia Appendix 2**

**Table of Contents**

**Figure S1.** Summary plot of risk of bias (RoB 2).

**Figure S2.** Subgroup forest (ISI by intervention functional category).

**Figure S3.** Subgroup forest (ISI by comparator type).

**Figure S4.** Subgroup forest (ISI by insomnia diagnosis).

**Figure S5.** Subgroup forest (ISI by economic classification).

**Figure S6.** Subgroup forest (SE by intervention functional category).

**Figure S7.** Subgroup forest (SE by comparator type).

**Figure S8.** Subgroup forest (SE by insomnia diagnosis).

**Figure S9.** Subgroup forest (SE by economic classification).

**Figure S10.** Subgroup forest (SE by measurement tool).

**Figure S11.** Subgroup forest (TST by intervention functional category).

**Figure S12.** Subgroup forest (TST by comparator type).

**Figure S13.** Subgroup forest (TST by insomnia diagnosis).

**Figure S14.** Subgroup forest (TST by economic classification).

**Figure S15.** Subgroup forest (TST by measurement tool).

**Figure S16.** Subgroup forest (PSQI by intervention functional category).

**Figure S17.** Subgroup forest (PSQI by comparator type).

**Figure S18.** Subgroup forest (PSQI by insomnia diagnosis).

**Figure S19.** Subgroup forest (PSQI by economic classification).

**Figure S20.** Forest plot (SOL overall).

**Figure S21.** Forest plot (WASO overall).

**Figure S22.** Forest plot (DBAS overall).

**Figure S23.** Forest plot (ESS overall).

**Figure S24.** Subgroup forest (SOL measurement tool).

**Figure S25.** Subgroup forest (WASO measurement tool).

**Figure S26.** Funnel plot for small-study effects (SOL).

**Figure S27.** Funnel plot for small-study effects (WASO).

**Figure S28.** Funnel plot for small-study effects (DBAS).

**Figure S29.** Funnel plot for small-study effects (ESS).

**Figure S1 –** Risk-of-bias summary plot for 44 randomized controlled trials using the RoB 2 tool, illustrating the proportion of studies with low risk, some concerns, and high risk across each domain and overall.

**Figure S2 –** Subgroup forest plot of digital health interventions versus non-digital controls for insomnia severity stratified by intervention functional category. HKSJ: Hartung-Knapp-Sidik-Jonkman random-effects model.

**Figure S3 –** Subgroup forest plot of digital health interventions versus non-digital controls for insomnia severity stratified by comparator type. HKSJ: Hartung-Knapp-Sidik-Jonkman

**Figure S4 –** Subgroup forest plot of digital health interventions versus non-digital controls for insomnia severity stratified by insomnia diagnosis. HKSJ: Hartung-Knapp-Sidik-Jonkman

**Figure S5 –** Subgroup forest plot of digital health interventions versus non-digital controls for insomnia severity stratified by economic classification. HKSJ: Hartung-Knapp-Sidik-Jonkman random-effects model.

**Figure S6 –** Subgroup forest plot of digital health interventions versus non-digital controls for sleep efficiency (SE) stratified by intervention functional category. HKSJ: Hartung-Knapp-Sidik-Jonkman random-effects model.

**Figure S7 –** Subgroup forest plot of digital health interventions versus non-digital controls for sleep efficiency (SE) stratified by comparator type. HKSJ: Hartung-Knapp-Sidik-Jonkman random-effects model.

**Figure S8 –** Subgroup forest plot of digital health interventions versus non-digital controls for sleep efficiency (SE) stratified by insomnia diagnosis. HKSJ: Hartung-Knapp-Sidik-Jonkman random-effects model.

**Figure S9 –** Subgroup forest plot of digital health interventions versus non-digital controls for sleep efficiency (SE) stratified by economic classification. HKSJ: Hartung-Knapp-Sidik-Jonkman random-effects model.

**Figure S10 –** Subgroup forest plot of digital health interventions versus non-digital controls for sleep efficiency (SE) stratified by measurement tool. HKSJ: Hartung-Knapp-Sidik-Jonkman random-effects model.

**Figure S11 –** Subgroup forest plot of digital health interventions versus non-digital controls for total sleep time (TST) stratified by intervention functional categor. HKSJ: Hartung-Knapp-Sidik-Jonkman random-effects model.

**Figure S12 –** Subgroup forest plot of digital health interventions versus non-digital controls for total sleep time (TST) stratified by comparator type. HKSJ: Hartung-Knapp-Sidik-Jonkman random-effects model.

**Figure S13 –** Subgroup forest plot of digital health interventions versus non-digital controls for total sleep time (TST) stratified by insomnia diagnosis. HKSJ: Hartung-Knapp-Sidik-Jonkman random-effects model.

**Figure S14 –** Subgroup forest plot of digital health interventions versus non-digital controls for total sleep time (TST) stratified by economic classification. HKSJ: Hartung-Knapp-Sidik-Jonkman random-effects model.

**Figure S15 –** Subgroup forest plot of digital health interventions versus non-digital controls for total sleep time (TST) stratified by measurement tool. HKSJ: Hartung-Knapp-Sidik-Jonkman random-effects model.

**Figure S16 –** Subgroup forest plot of digital health interventions versus non-digital controls for Pittsburgh Sleep Quality Index (PSQI) stratified by intervention functional category. HKSJ: Hartung-Knapp-Sidik-Jonkman random-effects model.

**Figure S17 –** Subgroup forest plot of digital health interventions versus non-digital controls for Pittsburgh Sleep Quality Index (PSQI) stratified by comparator type. HKSJ: Hartung-Knapp-Sidik-Jonkman random-effects model.

**Figure S18 –** Subgroup forest plot of digital health interventions versus non-digital controls for Pittsburgh Sleep Quality Index (PSQI) stratified by insomnia diagnosis. HKSJ: Hartung-Knapp-Sidik-Jonkman random-effects model.

**Figure S19 –** Subgroup forest plot of digital health interventions versus non-digital controls for Pittsburgh Sleep Quality Index (PSQI) stratified by economic classification. HKSJ: Hartung-Knapp-Sidik-Jonkman random-effects model.

**Figure S20 –** Forest plot of digital health interventions versus non-digital controls for sleep onset latency (SOL). HKSJ: Hartung-Knapp-Sidik-Jonkman random-effects model.

**Figure S21 –** Forest plot of digital health interventions versus non-digital controls for wake after sleep onset (WASO). HKSJ: Hartung-Knapp-Sidik-Jonkman random-effects model.

**Figure S22 –** Forest plot of digital health interventions versus non-digital controls for Dysfunctional Beliefs and Attitudes about Sleep (DBAS). HKSJ: Hartung-Knapp-Sidik-Jonkman random-effects model.

**Figure S23 –** Forest plot of digital health interventions versus non-digital controls for Epworth Sleepiness Scale (ESS). HKSJ: Hartung-Knapp-Sidik-Jonkman random-effects model.

**Figure S24 –** Subgroup forest plot of digital health interventions versus non-digital controls for sleep onset latency (SOL) stratified by measurement tool. HKSJ: Hartung-Knapp-Sidik-Jonkman random-effects model.

**Figure S25 –** Subgroup forest plot of digital health interventions versus non-digital controls for wake after sleep onset (WASO) stratified by measurement tool. HKSJ: Hartung-Knapp-Sidik-Jonkman random-effects model.

**Figure S26 –** Funnel plot for assessment of small-study effects in studies reporting sleep onset latency (SOL).

**Figure S27 –** Funnel plot for assessment of small-study effects in studies reporting wake after sleep onset (WASO).

**Figure S28 –** Funnel plot for assessment of small-study effects in studies reporting Dysfunctional Beliefs and Attitudes about Sleep (DBAS).

**Figure S29 –** Funnel plot for assessment of small-study effects in studies reporting Epworth Sleepiness Scale (ESS).
