# Supplementary material for: Efficacy of Digital Health Interventions for Insomnia: Systematic Review and Meta-Analysis of Randomized Controlled Trials
Source: J Med Internet Res. 2026 Nov 13;28:e90905. doi: 10.2196/90905 (PMC13766658; doi:10.2196/90905)
Supplement: Supplementary file 4 [file jmir-v28-e90905-s006.docx]

**PRISMA-S Checklist**

| **Section/topic** | **#** | **Checklist item** | **Location(s) Reported** |
| --- | --- | --- | --- |
| **INFORMATION SOURCES AND METHODS** | | | |
| Database name | 1 | Name each individual database searched, stating the platform for each. | Methods: Search Strategy and Information Sources (p. 7). The following databases were searched individually: PubMed (via NCBI), Embase (via Elsevier), Cochrane Library (via Wiley), Web of Science (via Clarivate), Scopus (via Elsevier), CINAHL (via EBSCOhost), CNKI, CBM, VIP, and Wanfang. |
| Multi-database searching | 2 | If databases were searched simultaneously on a single platform, state the name of the platform, listing all of the databases searched. | Methods: Search Strategy and Information Sources (p. 8). No multi-database platform searching was performed; each database was searched individually. |
| Study registries | 3 | List any study registries searched. | Methods: Search Strategy and Information Sources (p. 8). [ClinicalTrials.gov](https://clinicaltrials.gov/) was searched as a supplementary source. |
| Online resources and browsing | 4 | Describe any online or print source purposefully searched or browsed (e.g., tables of contents, print conference proceedings, web sites), and how this was done. | Methods: Information Sources (p. 8) and Multimedia Appendix 1 (p. 11). In addition to the aforementioned databases and [ClinicalTrials.gov](https://clinicaltrials.gov/), we manually browsed the website of the American Academy of Sleep Medicine. |
| Citation  searching | 5 | Indicate whether cited references or citing references were examined, and describe any methods used for locating cited/citing references (e.g., browsing reference lists, using a citation index, setting up email alerts for references citing included studies). | Methods: Study Selection (pp. 7-8). Backward citation searching was performed by manually screening the reference lists of all included studies and relevant systematic reviews. Forward citation searching was conducted using Web of Science and Scopus. |
| Contacts | 6 | Indicate whether additional studies or data were sought by contacting authors, experts, | Methods: Search Strategies (p. 8). We did not contact study authors or experts for additional data. |

|  |  | manufacturers, or others. |  |
| --- | --- | --- | --- |
| Other methods | 7 | Describe any additional information sources or search methods used. | Methods: Search Strategies (p. 8). No additional search methods were used. |
| **SEARCH STRATEGIES** | | | |
| Full search  strategies | 8 | Include the search strategies for each database and information source, copied and pasted exactly as run. | Supplementary Methods in Multimedia Appendix 1. Full search strategies for all 10 databases and the AASM website are provided. |
| Limits and  restrictions | 9 | Specify that no limits were used, or describe any limits or restrictions applied to a search (e.g., date or time period, language, study design) and provide justification for their use. | Methods: Search Strategies (pp. 7-8). No language restrictions were applied. Searches were conducted from database inception to July 2026. No other search limits or restrictions (e.g., study design, publication type) were applied as database search filters. Study design was restricted to randomized controlled trials and controlled clinical trials at the eligibility stage, not as a search limit. |
| Search filters | 10 | Indicate whether published search filters were used (as originally designed or modified), and if so, cite the filter(s) used. | Methods: Search Strategy (p. 8). No published search filters (e.g., for study design or topic) were used in the search strategies, either as originally designed or in modified form. |
| Prior work | 11 | Indicate when search strategies from other literature reviews were adapted or reused for a substantive part or all of the search, citing the previous review(s). | Methods: Search Strategy (p. 8). The search strategy was developed de novo and was not adapted or reused from prior systematic reviews. |
| Updates | 12 | Report the methods used to update the search(es) (e.g., rerunning searches, email alerts). | Methods: Search Strategies (p. 7). The search was updated by rerunning the full search strings in all identified databases until July 2026. |

| Dates of  searches | 13 | For each search strategy, provide the date when the last search occurred. | Methods: Search Strategies (p. 7). The search was conducted from database inception to July 2026. |
| --- | --- | --- | --- |
| **PEER REVIEW** | | | |
| Peer review | 14 | Describe any search peer review process. | Methods: Search Strategies (p. 8). The search strategies were developed by the review authors and were not subjected to formal external peer review, such as that performed using the PRESS (Peer Review of Electronic Search Strategies) guideline. |
| **MANAGING RECORDS** | | | |
| Total Records | 15 | Document the total number of records identified from each database and other information sources. | Results: Study Selection (pp. 13-14). Total records identified are reported in the PRISMA flow diagram (Figure 1). |
| Deduplication | 16 | Describe the processes and any software used to deduplicate records from multiple database searches and other information sources. | Methods: Study Selection (pp. 7-8). All retrieved records were imported into EndNote 21 for screening, and duplicate records were removed using both automated and manual processes. |

PRISMA-S: An Extension to the PRISMA Statement for Reporting Literature Searches in Systematic Reviews Rethlefsen ML, Kirtley S, Waffenschmidt S, Ayala AP, Moher D, Page MJ, Koffel JB, PRISMA-S Group.

Last updated February 27, 2020.
